# Morphological reassessment of the movable calcar of delphacid planthoppers (Hemiptera: Fulgoromorpha: Delphacidae)

**DOI:** 10.1038/s41598-021-01771-9

**Published:** 2021-11-16

**Authors:** Darya Markevich, Marcin Walczak, Oleg Borodin, Jacek Szwedo, Jolanta Brożek

**Affiliations:** 1 State Scientific and Production Amalgamation The Scientific and Practical Center for Bioresources, Laboratory of Terrestrial Invertebrates, National Academy of Sciences of Belarus, 27, Akademicheskaya Str., 220050 Minsk, Belarus; 2 Faculty of Natural Sciences, Institute of Biology, Biotechnology and Environmental Protection, University of Silesia in Katowice, 9, Bankowa St., 40007 Katowice, Poland; 3 Institute of Systematic Biology, Daugavpils University, 13 – 229 Vienības Street, Daugavpils, 5401 Latvia; 4 Laboratory of Evolutionary Entomology and Museum of Amber Inclusions, Department of Invertebrate Zoology and Parasitology, Faculty of Biology, University of Gdańsk, 59, Wita Stwosza St., 80309 Gdańsk, Poland

**Keywords:** Evolution, Zoology

## Abstract

This study presents the morphology of calcar in adult Delphacidae based on representatives of the genera *Ugyops* Guérin-Meneville, 1834, *Notuchus* Fennah, 1969 (Ugyopini), *Asiraca* Latreille, 1798 (Asiracini), *Kelisia* Fieber, 1866, (Kelisini), *Stenocranus* Fieber, 1866 (Stenocranini), *Chloriona* Fieber, 1866, *Megadelphax* Wagner, 1963, *Muellerianella* Wagner, 1963, *Javesella* Fennah, 1963, *Conomelus* Fieber, 1866, *Euconomelus* Haupt, 1929, *Hyledelphax* Vilbaste, 1968, *Stiroma* Fieber, 1866, *Struebingianella* Wagner, 1963 and *Xanthodelphax* Wagner, 1963 (Delphacini). We used SEM electron microscopy, to define seven types of calcar structure (Types 1, 2, 5, 6, 7, 8, and 9) based on combinations of characters including shape, number of teeth and differentiation of sensory structures in species from fifteen genera. Additionally, two other types (Types 3 and 4) were determined based on the calcar descriptions from previous studies. Similarities and differences in calcar structure and function were discussed and emerging relationships between planthopper species and their particular habitats were indicated.

## Introduction

The planthopper family Delphacidae Leach, 1815 (Hemiptera, Fulgoromorpha: Fulgoroidea) is a relatively large and widely distributed group of opophagous insects feeding on plants, mainly on phloem. Delphacids comprise 2217 species in 427 genera and six subfamilies worldwide^1,2^. These planthoppers are associated mostly with monocotyledonous plants, especially grasses and sedges^3–5^. Some species also feed on horsetails and even mosses^6,7^, often living in wet—or at least mesic—situations^8,9^; endemic delphacids in Hawai’i have evolved to feeding onto many dicotyledons taxa^10^.

The economic importance of planthoppers is well documented^11^. The family contains at least 55 species that feed on economic plants including major pests of five of the top ten major world food crops, such as rice, sugarcane, maize, taro, and cereals. For example, *Nilaparvata lugens* (Stål, 1854), *Sogatella furcifera* (Horváth, 1899) and *Laodelphax striatellus* (Fallén, 1826) cause serious damage to rice paddies in south-eastern Asia^12–15^.

Delphacidae are mostly small or medium-sized insects, with most species between 2 and 4 mm in size (ranging from ~ 1.5 to  ~ 10.0 mm); their compact bodies with usually short head and distinct keels are mainly cylindrical in cross-section. The presence of a movable calcar—the spur is the most charismatic and diagnostic character of the family, by which delphacids are easily distinguished from other planthoppers and similar insect families (viz. the other planthoppers and cicadomorphans). The calcar is located at the tip of the metatibia and base of the metatarsus, and it is articulated with the hind tibia^16,17^. Other distinctive characters include: basitmetarsomere and midmetarsomere with a row of apical teeth; ovipositors enormously long dividing the sternites right to the base of the abdomen; the tegmina membranous or somewhat thickened (especially when brachypterous), and held tent-like; they may be clear or patterned. Dimorphism is often expressed with adults either long-winged (macropterous, or dispersal forms, with fully developed flying wings) or short-winged (brachypterous, adult wings not functional for flying) within a single population of a species^9,18^. The calcar and its features are used to differentiate major groups within the family.

The classification and internal subdivisions of Delphacidae have been subjects of disputes. The division into two subfamilies (Asiracinae Motschulsky, 1863 and Delphacinae Leach, 1815) proposed by Muir^19,20^, followed by Metcalf^21^, subdivided Delphacinae into three tribes: Alohini Muir, 1915, Tropidocephalini Muir, 1915 and Delphacini Leach, 1815 (= Areopini Metcalf, 1938). Nevertheless, other opinions were also presented. Haupt^22^ suggested division into 4 subfamilies (Asiracinae, Tropidocephalinae Muir, 1915, Criomorphinae Kirkaldy, 1910 (= Megamelinae Haupt, 1929 see Nast^23^), and Delphacinae Leach, 1815). Later, Wagner^24^ proposed a division with subfamilies: Asiracinae, Kelisiinae Wagner, 1963, Jassidaeinae Wagner, 1963, Stirominae Wagner, 1963, Achorotilinae Wagner, 1963, Delphacinae, Chlorioninae Wagner, 1963, Stenocraninae Wagner, 1963, and Megamelinae. Vilbaste^25^ recognized the subfamily Saccharosydninae Vilbaste, 1968. Finally, Fennah^26^ divided the Asiracinae into two tribes (Asiracini Motschulsky, 1863 and Ugyopini Fennah, 1979). Those proposals were critically tested and verified with a detailed morphological cladistic analysis by Asche^27^. He distinguished 5 clades (subfamilies) within Delphacidae: paraphyletic Asiracinae and monophyletic ones: Kelisiinae, Stenocraninae, Plesiodelphacinae Asche, 1985^27^ and Delphacinae; later, he added a small subfamily Vizcayinae Asche, 1990 (as the sixth clade) into the family^28^. Similar subdivisions were presented by Emeljanov^29^ after morphological studies on larval stages, with proposed subfamilies Ugyopinae Fennah, 1979 (with tribes Neopunanini Emeljanov, 1995, Eodelphacini Emeljanov, 1995 and Ugyopini), Asiracinae (with tribes Tetrastreini Emeljanov, 1995, Platysystatini Emeljanov, 1995, Asiracini and Idiosystatini Asche, 1985b) and Delphacinae (with 7 tribes: Vizcayini Asche, 1990, Kelisiini Wagner, 1963, Stenocranini Wagner, 1963, Plesiodelphacini Asche, 1985, Tropidocephalini Muir, 1915, Saccharosydnini Vilbaste, 1968 and Delphacini Leach, 1815). Later, Emeljanov^30^ recognized Kelisiinae, Sternocraninae, Saccharosydninae, and Tropidocephalinae as subfamilies. Hamilton^31^, following the general scheme of Emeljanov^29^, proposed to treat Kelisiini as a subtribe of Stenocranini, and Saccharosydnini (Delphacinae) as a subtribe of Tropidocephalini.

The first advances based on molecular data based on 504 bp of cytochrome oxidase I^32^ and 352 bp of 12S rDNA^33^ employed limited taxonomic and data sampling but provided some insight into higher level relationships within Delphacidae. Later, Urban et al.^4^ tested the phylogenetic proposals of Delphacidae based on DNA nucleotide sequence data from four genetic loci (18S rDNA, 28S rDNA, wingless and cytochrome oxidase I) and 132 coded morphological characters. The received topology generally supported higher classifications of Delphacidae proposed by Asche, Emeljanov and Hamilton, and suggested a rapid diversification of the Delphacini associated with host shifts to, and within, Poaceae, and specifically from C3 to C4 grasses^4^. More molecular studies explored the phylogeny of the delphacid subfamily Delphacinae based on nuclear ribosomal and mitochondrial DNA sequences of four genetic loci (16S rDNA, 28S rDNA, COI and Cyt b)^5,34^. Recently, mitogenomes of some Delphacidae were sequenced and used to reconstruct the phylogeny^35–38^. Additional morphological data on the ovipositor were presented by Wallner & Bartlett^39^.

The family Delphacidae is commonly accepted as a monophyletic unit, based on unique synapomorphy (the presence of a metatibial calcar). The origin of the family remains doubtful; several previous studies based on either morphological^16,27,40,41^ or molecular^1,4,42–44^ evidence suggested that Delphacidae might have arisen from within the planthopper family Cixiidae—resulting in the paraphyletic status of the latter.

The sister group of Delphacidae—the Cixiidae Spinola, 1839 is known in the fossil record from the Lower Cretaceous, Barremian^45,46^, while the oldest undoubted fossil Delphacidae are much younger, from the Middle Eocene Baltic amber^47^ and then from Miocene deposits of Russia, and Miocene fossil resins^48,49^. Fossils from early Eocene deposits of Green River^50–53^, Late Eocene/Early Oligocene of Russian Far East^53^ and Late Oligocene of Rott^54^ cannot be unequivocally ascribed to Delphacidae.

The calcar is an ancient feature, which occurs on legs of many insects and differs from setae in being multicellular in origin; in some species it has been lost or modified over evolutionary time to suit the adaptive needs of different groups^55,56^. The size and shape of this structure is highly variable, and it seems to have been put to a wide variety of uses (like the blades on a Swiss army knife). However, calcar’s exact function remains unclear because only few studieshave dealt with related structures^57–61^. This reservation also applies to a specialized spur of Delphacidae—the calcar. It is generally believed that the calcar is used to assist in jumping; however, it is morphologically disparate within delphacid subfamilies. The features of calcar were previously studied under light microscopy, and the results were presented by Metcalfe^62^, Wilson & McPherson^63^, Asche^27^, Liang^64^ and Bartlett & Webb^65^. The reassessment of calcar characters can provide additional data giving insights into the differentiation and morphological disparity of Delphacidae subfamilies; potentially it can indicate adaptation to the structure and surface of their host plants. Additionally, the knowledge of calcar structures will be of great importance for interpreting morphological data available from fossils and tracing evolutionary changes. Therefore, we examined the calcar using SEM to describe new features and possible adaptive characters for moving on different host-plant surfaces.

## Material and methods

The studied materials come from the collections of the Upper Silesian Museum in Bytom (USMB) (*Notuchus* and *Ugyops*), the collection of the Zoology Research Team, University of Silesia in Katowice (DZUS) (species of several different genera *e.g*., *Asiraca, Kelisia*, *Chloriona*, *Megadelphax*, *Conomelus*, *Euconomelus*, *Muellerianella*, *Hyledelphax*, *Stiroma*, *Struebingianella*, *Xanthodelphax*) and the collection of the Laboratory of Terrestrial Invertebrates (LTIB)—State Scientific and Production Amalgamation The Scientific and Practical Center for Bioresources, National Academy of Sciences of Belarus (mainly *Stenocranus* and *Javesella*).

SEM examinations were conducted in the Laboratory of Scanning Microscopy of the Institute Biology, Biotechmology and Environmental Protection, the University of Silesia in Katowice. The dry material (20 species and 80 specimens) was cleaned in an ultrasonic cleaner for several seconds. Then the specimens were subsequently dehydrated in an ascending ethanol series (30, 50, 70, 80, 96, and 100%, for 10 min in each concentration with three 100% ethanol changes) and were air-dried at room temperature for 12 h. The samples were mounted on aluminium stubs with double-sided adhesive carbon tape and sputter-coated in a Pelco SC-6 sputter coater (Ted Pella Inc., Redding, CA, USA) with a thin film of gold. After processing, samples were imaged by the Phenom XL scanning electron microscope. For taking images, an eucentric sample holder was used to allow the sample to freely move, including rotate and tilt, to show all surfaces. The calcar usually protrudes from the tibia, so samples could be rotated and imaged for all elements in detail in SEM.

As previously noted, the metatibial apical movable spur of the Delphacidae, i.e., the calcar, is defined as a special structure usually flattened, foliaceous, and bearing a row of black-tipped teeth on the posterior (trailing) margin or spine-like shaped and not toothed^9^_._ The historical background of the calcar studies and descriptions are presented in Table S1. Terms of surface sculpturing have been used according to a web available glossary^66^.

Taxa examined:

Asiracinae: Asiracini—*Asiraca clavicornis* Latreille, 1802;

Asiracinae: Ugyopini—*Notuchus linnavuorii* Gębicki & Walczak, 2021; *Ugyops inermis* Distant, 1920; *Ugyops nemestrinus* Fennah, 1969; *Ugyops taranis* Fennah, 1964;

Kelisiinae—*Kelisia praecox* Haupt, 1935;

Delphacinae: Delphacini—*Chloriona smaragdula* (Stål, 1853); *Conomelus anceps* (Germar, 1821); *Euconomelus lepidus* (Boheman, 1847); *Hyledelphax elegantulus* (Boheman, 1847)*; Javesella* (*Javesella*) *pellucida* (Fabricius, 1794); *Megadelphax sordidula* (Stål, 1853) *Muellerianella brevipennis* (Boheman, 1847); *Stiroma affinis* Fieber, 1866; *Struebingianella lugubrina* (Boheman, 1847); *Xanthodelphax straminea* (Stål, 1858); Stenocraninae: *Stenocranus fuscovittatus* (Stål, 1858); *Stenocranus major* (Kirschbaum, 1868).

## Results

**Figure 1:**
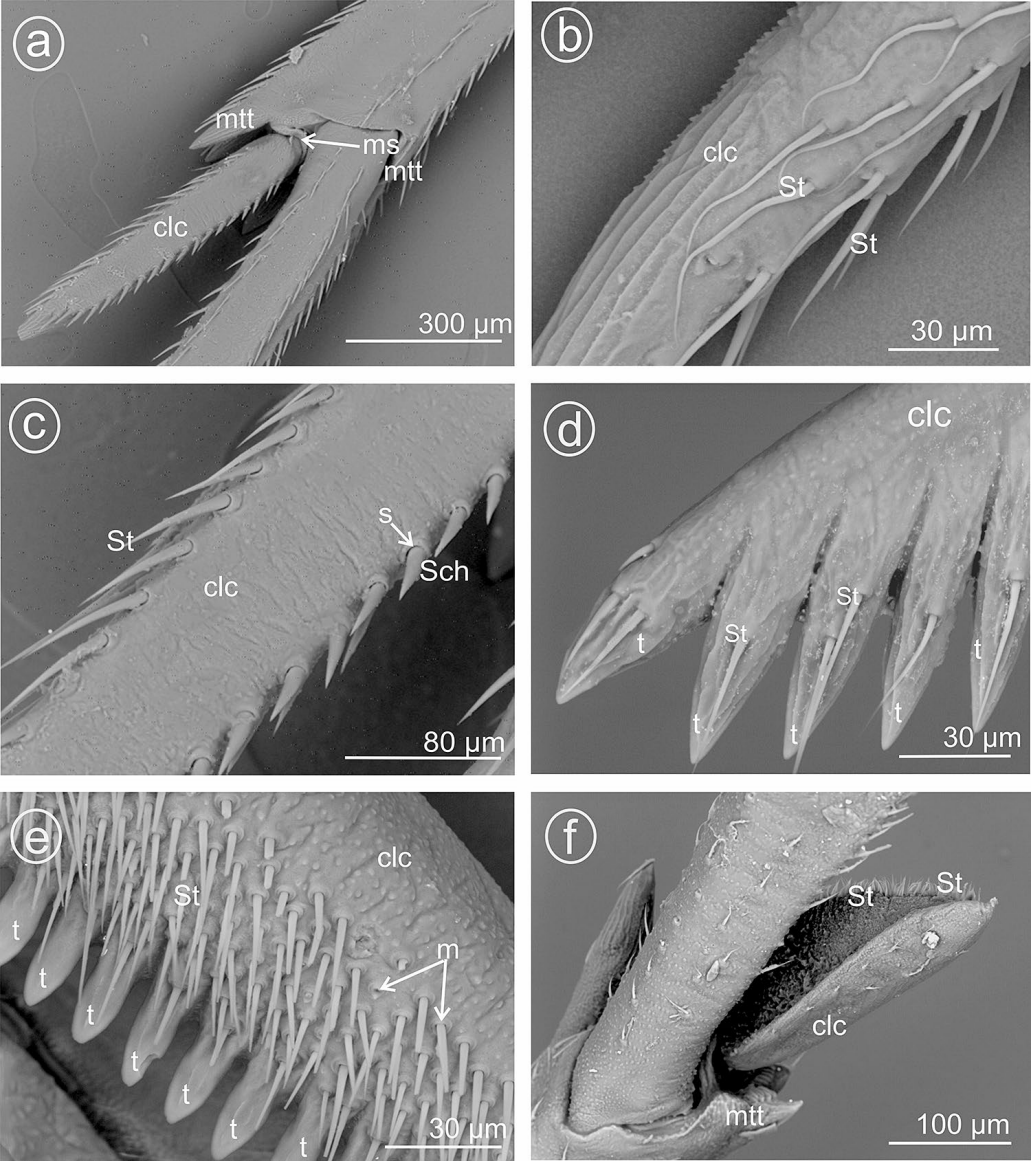
Calcar types in Delphacidae, (**a**) *Ugyops taranis*, membranous connection (ms) of calcar (clc), (**b**) *Asiraca clavicornis*, narrow spike calcar (clc) with sensilla trichoidea (St), (**c**) *Ugyops taranis*, triangular calcar with sensilla trichoidea (St) and chaetica(Sch), (**d**) *Kelisia praecox*, calcar (clc) with several large teeth (t) with sensilla trichoidea (St), (**e**) *Chloriona smaragdula*, calcar (clc) with numerous smaller teeth (t) and with sensilla trichoidea (St), (**f**) *Stiroma affinis*, calcar (clc) with numerous sensilla trichoidea (St), *m* flexible membrane, *ms* membranous connection of the calcar, *mtt* metatibial apical row of teeth, *s* socket.

The present study distinguished nine types of the calcar structure based on the combination of characters of its shape, number of teeth and differentiation of sensory structures. The calcar (clc) (Fig. 1a–f) as a whole is a movable structure, with a membranous connection (ms) at the apex of metatibia, under the metatibial crown (mtt) of a few teeth (apical row of teeth). The sensory structures were identified as sensilla trichoidea (St)—larger setae, hair-like, and sensilla chaetica (Sch)—shorter and stouter setae. Both sensilla belong to a group of the mechanosensilla, which detect most of the tactile sensations perceived by the insect. Sensilla trichoidea (St) are characteristic in a more flexible stem as opposed to sensilla chaetica, which are provided with a stiffer, slightly grooved and acutely terminating stem. Both types of sensilla are embedded in flexible sockets (s), with a flexible membrane (m) surrounding the hair base. The distinguished types of calcar are listed below and in Table 1.

**Table 1:** Summary of the calcar morphology based on SEM images.

Calcar type	Shape	Apex	Cross section	Armature	Sensilla trichoidea	Sensilla chaetica
Type 1	Awl-shaped (subulate), elongated, not flattened	Slightly costate sculpturing	Rounded	None	Irrregular, on outer side	–
Type 2	Subulate, angular	Distinct costate sculpturing	Subtriangular	None	Long, in rows	Stout, in rows
Type 3	Subulate	Apical tooth	Terete	Sparsely dentate	Irregularly interspersed between the bases of teeth	Above the bases of teeth
Type 4	Cultrate	Apical tooth	Oval	Teeth large, sparse	Two rows along the outer margin, scarcely dispersed	Short, placed at the base of each internal tooth
Type 5	Tectiform	Apical tooth	Subtriangular	Sparsely dentate	Singular long sensilla placed at subbasal of the teeth	–
Type 6	Tectiform	Apical tooth	Subtriangular to sickle-shaped	Densely dentate	Sensilla in narrow row	–
Type 7	Tectiform	Apical tooth	Subtriangular to sickle-shaped	Densely denticulate	Wide rows of dense long sensilla	–
Type 8	Tectiform	Apical tooth	Triangular to sickle-shaped in cross-section	Densely denticulate with bristly mechanosensilla	Brush of sensilla along with the teeth	–
Type 9	Tectiform	Apical tooth	Sickle-like to almost flat	Edentate	Wide rows of long sensilla	–

### Type 1: calcar awl-shaped (subulate), elongated, not flattened

**Figure 2:**
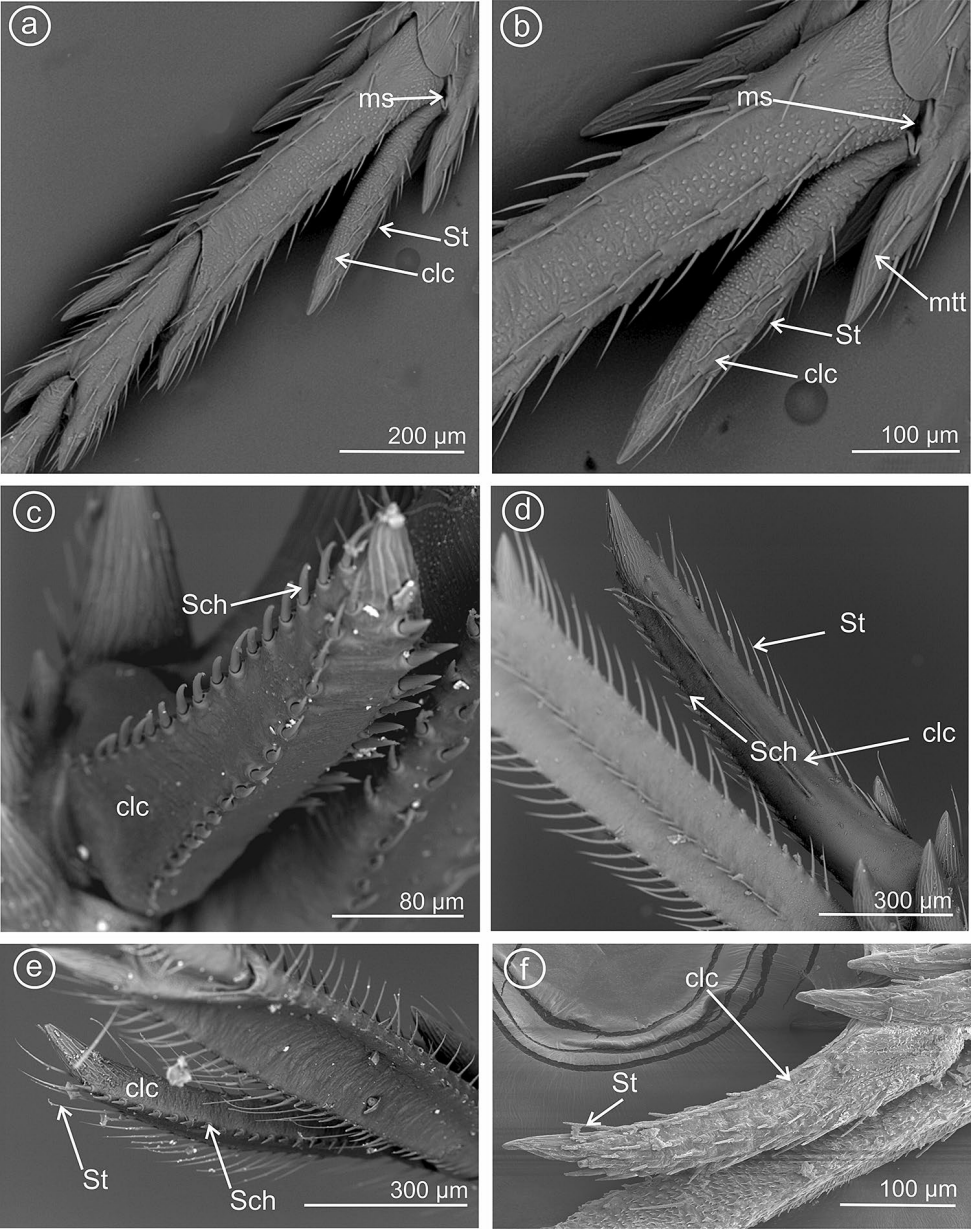
Shape of the calcar, dorsal view, (**a**,**b**) *Asiraca clavicornis*, (**c**) *Ugyops taranis*, (**d**) *U. nemestrinus*, (**e**) *U. inermis*, (**f**) *Notuchus linnavuorii*, *St* sensilla trichoidea, *Sch* sensilla chaetica, *clc* calcar, *ms* membranous connection, *mtt* metatibial apical row of teeth.

The calcar (clc) is similar to a long and narrow awl with convex surfaces, bearing several sensilla trichoidea. This type is present in species of the subfamily Asiracinae. In *Asiraca clavicornis* (Asiracini), the calcar is in the form of an elongated awl, about twice as long as metatibial apical teeth (apical row in the form of an open crown-like wreath). The apex has slightly costate sculpturing. The calcar is rounded in cross-section. Several irregularly arranged sensilla trichoidea (St) are located on the outer side (Fig. 2a,b).

### Type 2: calcar subulate, angular

In the representatives of the tribe Asiracine, the Ugyopini’s calcar is strongly spinose, subtriangular in cross-section, about three times as long as teeth of apical row; calcar internal (adplantar) side and dorsal (explantar) edges are delineated by rows of long sensilla trichoidea and stout sensilla chaetica. In *Ugyops taranis* (Fig. 2c), three rows of short and stout sensilla chaetica are visible. In *Ugyops nemestrinus* (Fig. 2d) and *Ugyops inermis* (Fig. 2e), there are two rows of long sensilla trichoidea (h) and one row of short stout sensilla chaetica (Sch) on the surface. The stout sensilla chaetica resemble sharp teeth. In *Notuchus linnavuorii* (Fig. 2f), the calcar is more subulate with external (explantar) surface convex and internal (adplantar) surface slightly concave; external (explantar) surface covered with shortened sensilla trichoidea (St). Inthree mentioned *Ugyops* species, the costate sculpturing of the calcar apex is deeper than that in *Asiraca clavicornis*.

### Type 3: calcar subulate,sparsely dentate

In *Vizcaya* Muir, 1917, and *Neovizcaya* Liang, 2002, the calcar is elongated, terete in cross-section, but a distinct row of 8–12 large teeth is present at the adplantar margin (Table S1). Bases of sensilla chaetica are placed above the bases of these teeth. A few sensilla trichoidea are irregularly interspersed between the bases of teeth (^64^: Fig. 18).

### Type 4: calcar cultrate, with large sparse teeth

Superficially similar to Type 3, the calcar is elongate, cultrate, oval in cross-section, convex on the adplantar and explantar sides, with a row of 4–8 large conical teeth along the inner margin; two rows of a few scarcely dispersed setae (sensilla trichoidea) along the outer margin, sensilla chaetica as short setae placed at the base of each internal tooth (^19,27,67^, viz.^27^, Figs. 294–305) (Table S1). Muir^19^ used this pattern of calcar to define tribe Alohini Muir, 1915 (in this paper comprising mostly endemic to Hawai’i and Pacific Islands genera: *Aloha* Kirkaldy, 1904, *Dictyophorodelphax* Swezey, 1907, *Leialoha* Kirkaldy, 1910, *Nesodryas* Kirkaldy, 1908 (subgenera *Nesodryas* and *Nesothoe* Kirkaldy, 1908), *Nesorestias* Kirkaldy, 1908, *Nesosydne* Kirkaldy, 1907, *Proterosydne* Kirkaldy, 1907); later Asche^27^ (p.310, see also Asche^10^: 369–370) synonymized Alohini under Delphacini.

### Type 5: calcar tectiform, sparsely dentate

**Figure 3:**
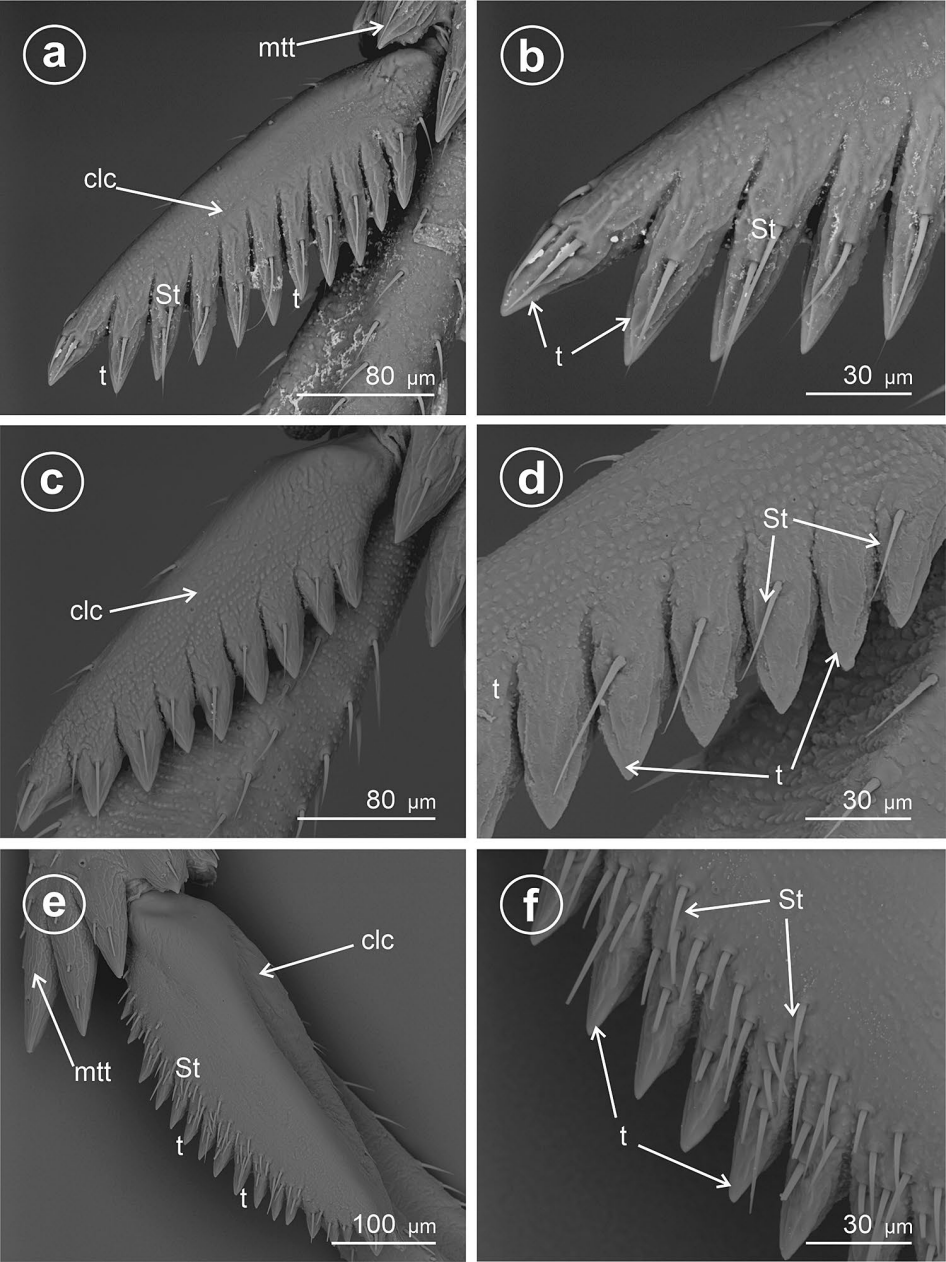
Dorsal view of calcar, (**a**,**b**) *Kelisia praecox*, (**c**,**d**) *Conomelus anceps*, (**e**,**f**) *Megamelus notulus*, *St* sensilla trichoidea, *clc* calcar, *mtt* metatibial apical row of teeth, *t* teeth.

**Figure 4:**
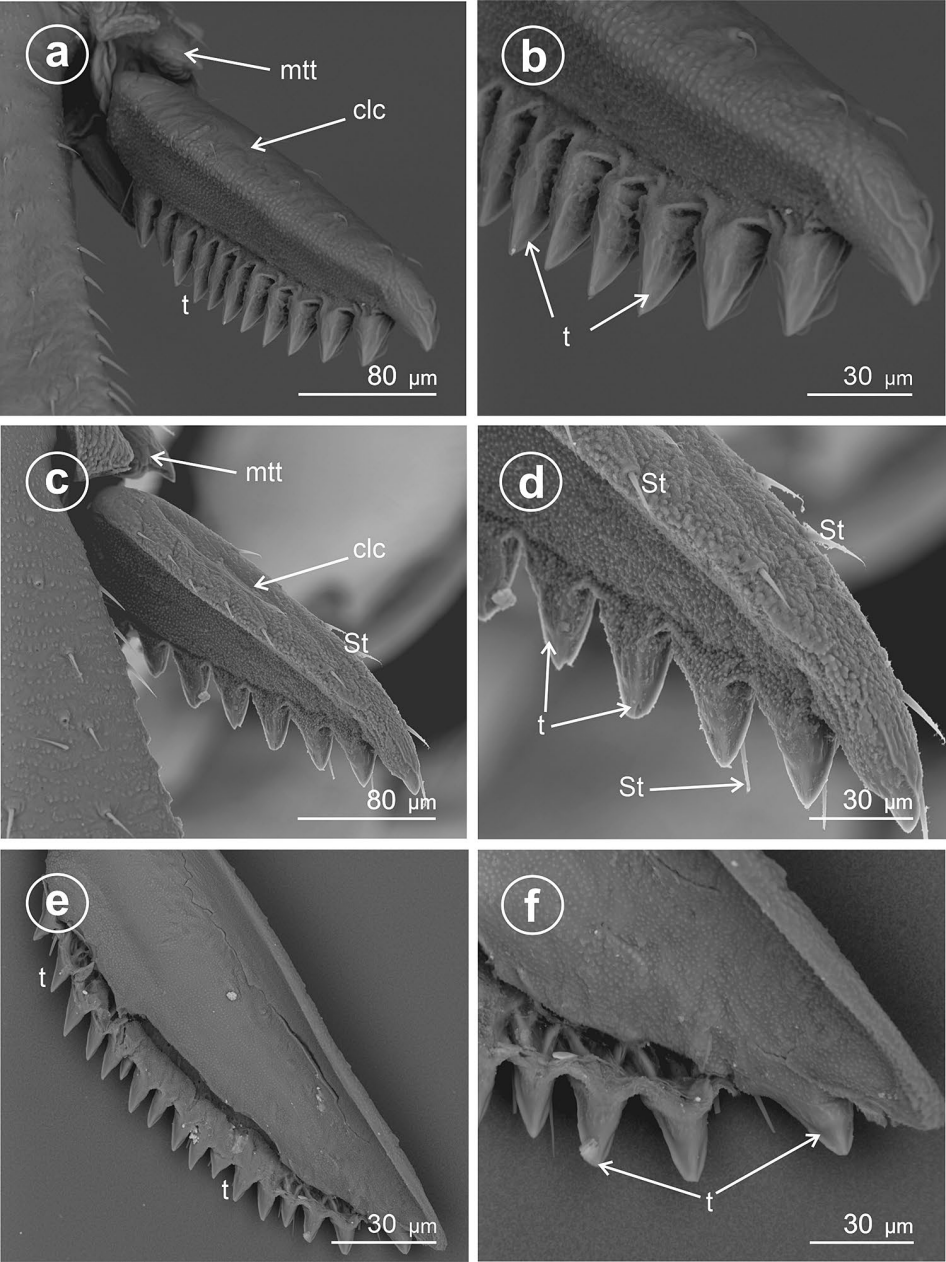
Ventral view of calcar, (**a**,**b**) *Kelisia praecox,* (**c**,**d**) *Conomelus anceps*, (**e**,**f**) *Megamelus notula*, *St* sensilla trichoidea, *clc* calcar, *mtt* metatibial apical row of teeth, *t* teeth.

Here, the edges (ventrad adplantar and dorsad adplantar) of calcar sloping downwards on two sides from a raised external, explantar margin. The ventral adplantar margin with a variable number (more than 8) of large distinct immovable conical teeth (t) (from 9 to 11 teeth, including the apical one), with subbasal, long sensilla trichoidea (St) (Fig. 3a–f). The dorsal adplantar margin is smooth and bent to the ventral side. Adplantar surface of calcar concave, in the form of a wide and shallow groove (Fig. 4a–f). The size and shape of calcar differ in the studied species.

In *Kelisia praecox* (Fig. 3a,b) (Kelisiinae) the ventral adplantar margin bears 11 long and thin conical teeth (including the apical tooth) and on the surface of each tooth there is one or two sensilla trichoidea (St). The remainder of calcar surface with a sparse sensilla (St).

In *Conomelus anceps* (Delphacinae: Delphacini) the ventral adplantar margin bears 9 short and stout teeth (Fig. 3c,d) which are shorter than those in *Kelisia praecox*. The surface of each tooth possesses one sensilla trichoidea (St). The remainder of calcar surface has a sparse sensilla (St) (Fig. 3c,d).

### Type 6: calcar tectiform, densely dentate

The calcar is subtriangular to sickle-shaped in cross-section, dorsal edge less noticeable. The ventral adplantar margin bears more than a dozen small teeth, covered with more or less dense mechanosensilla (St). The adplantar side of calcar lacks sensilla.

In *Megamelus notulus* (Germar, 1830) (Delphacinae: Delphacini) the adplantar margin bears 19 small teeth (Fig. 3e,f). The surface of each tooth possesses two rows of the sensilla trichoidea (St) above the teeth. The remainder of the calcar surface has a sparse mechnosensilla (St).

**Figure 5:**
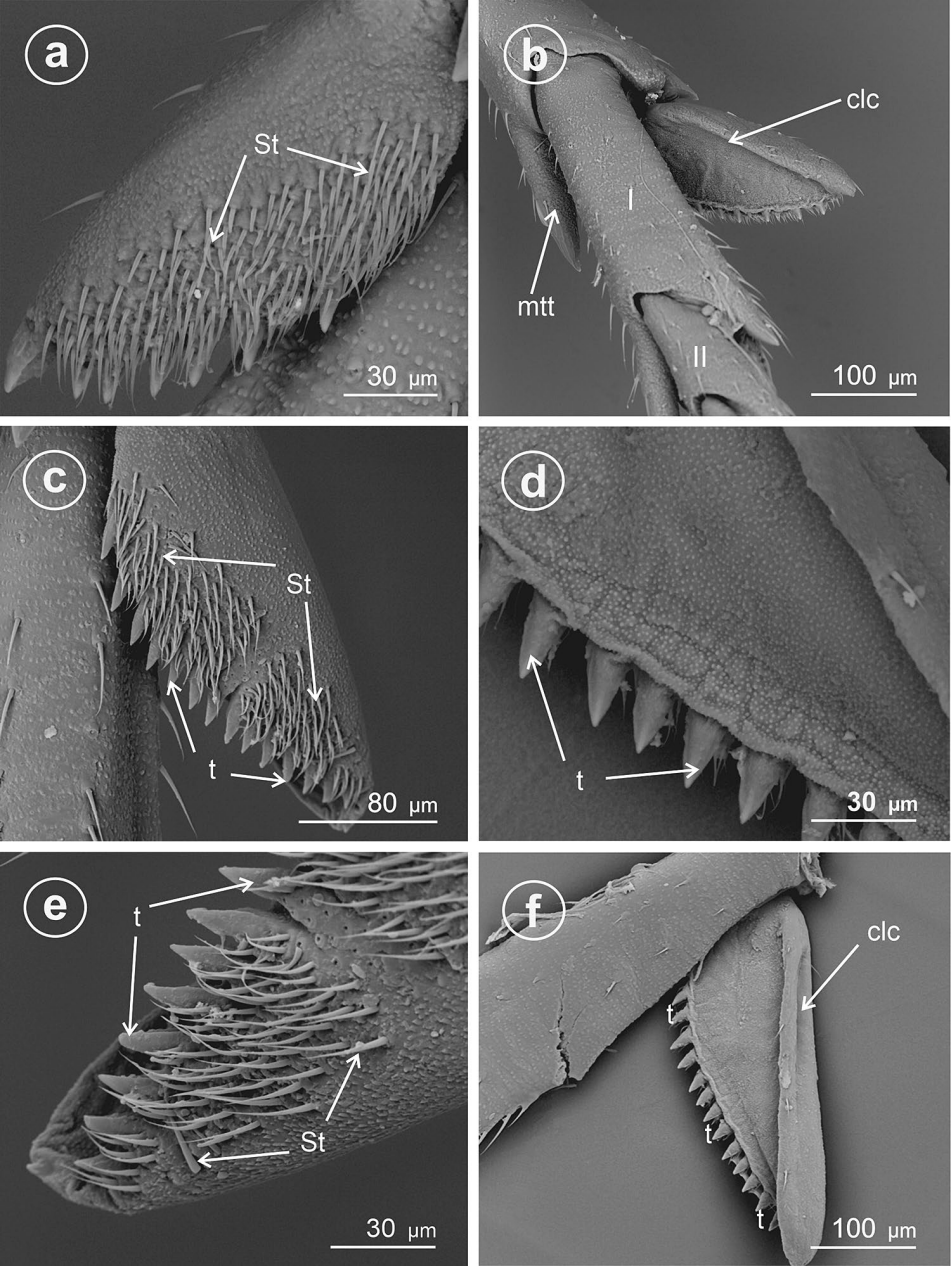
Dorsal and ventral view of calcar, (**a**,**c**,**e**) dorsal view, (**b**,**d**,**f**) ventral view. (**a**,**b**) *Euconomelus lepidus,* (**c**–**f**) *Muellerianella brevipennis*, *St* sensilla trichoidea, *clc* calcar, *mtt* metatibial apical row of teeth, *t* teeth.

In *Euconomelus lepidus* (Delphacinae: Delphacini) (Fig. 5a,b) the adplantar margin bears about 20 small teeth. The surface of each tooth and remainder of the adplantar slope are covered by abundant mechnosensilla (St), forming a brush hiding the teeth at the adplantar slope of calcar. The calcar adplantar surface bears no sensilla (Fig. 5b).

In *Muellerianella brevipennis* (Delphacinae: Delphacini) (Fig. 5c–f the adplantar margin bears 15 small teeth. The surfaces of each tooth and adplantar slope are covered by abundant mechanosensilla (St); however, the tips of the teeth are free of sensilla. These sensilla form a brush on the calcar edge. The inner surface of calcar bears no sensilla (Fig. 5d,f).

**Figure 6:**
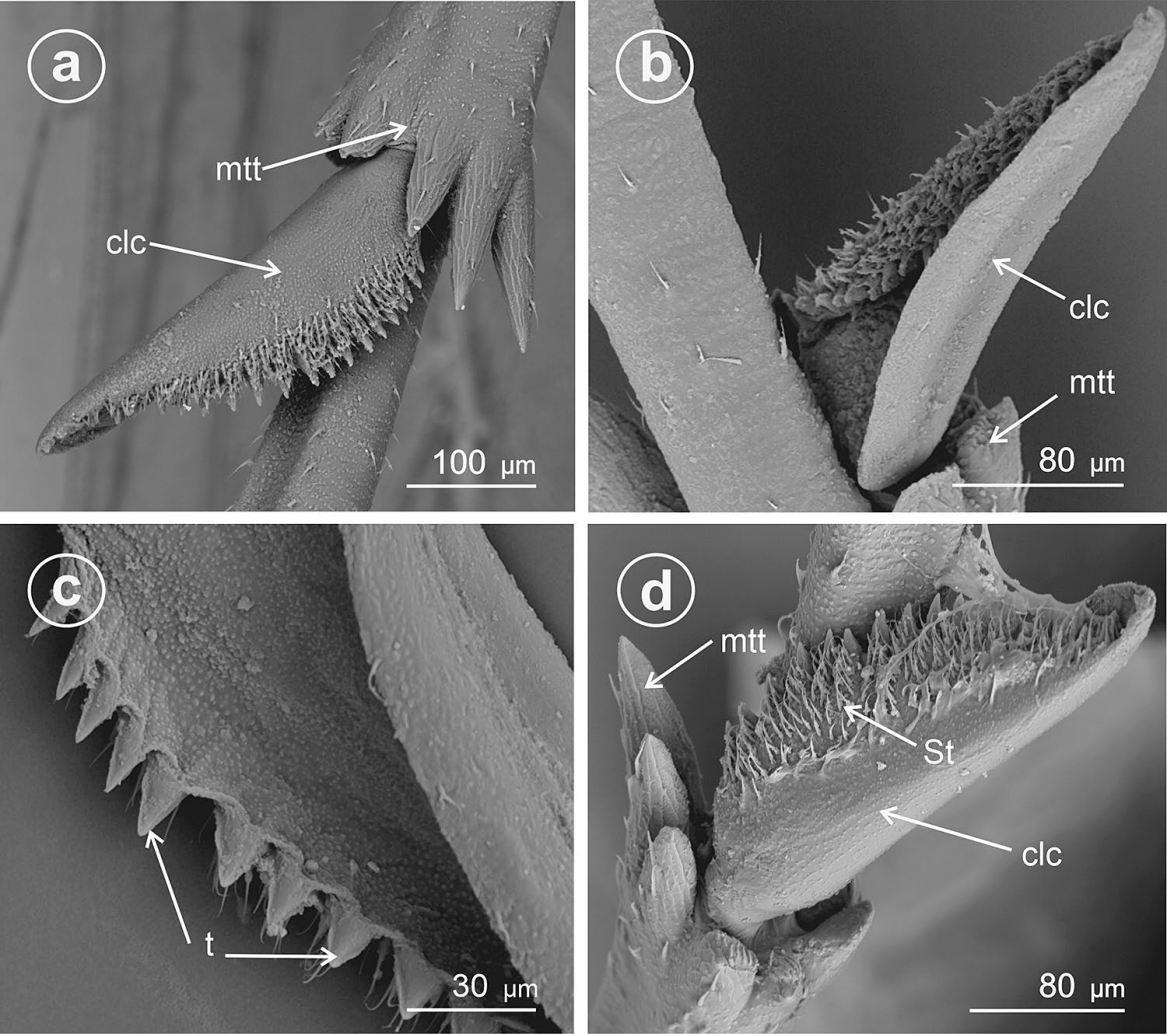
Dorsal and ventral view of calcar, (**a**,**d**) dorsal view, (**b**,**c**) ventral view. (**a**–**c**) *Javesella pellucida*, (**d**) *Laodelphax striatellus*, *St* sensilla trichoidea, *clc* calcar, *mtt* metatibial apical row of teeth, *t* teeth.

In *Javesella pellucida* (Fig. 6a–c) and *Laodelphax striatellus* (Fig. 6 d) (Delphacinae: Delphacini) the adplantar margin bears 20–24 small teeth (t) and several rows of the sensilla trichoidea (St).

### Type 7: calcar tectiform, densely denticulate

Similarly to Type 4, it is subtriangular to sickle-shaped in cross-section. The ventral adplantar margin bears 20 or more small teeth, and rows of densely packed mechanosensilla (St). The adplantar side of calcar lacks sensilla.

**Figure 7:**
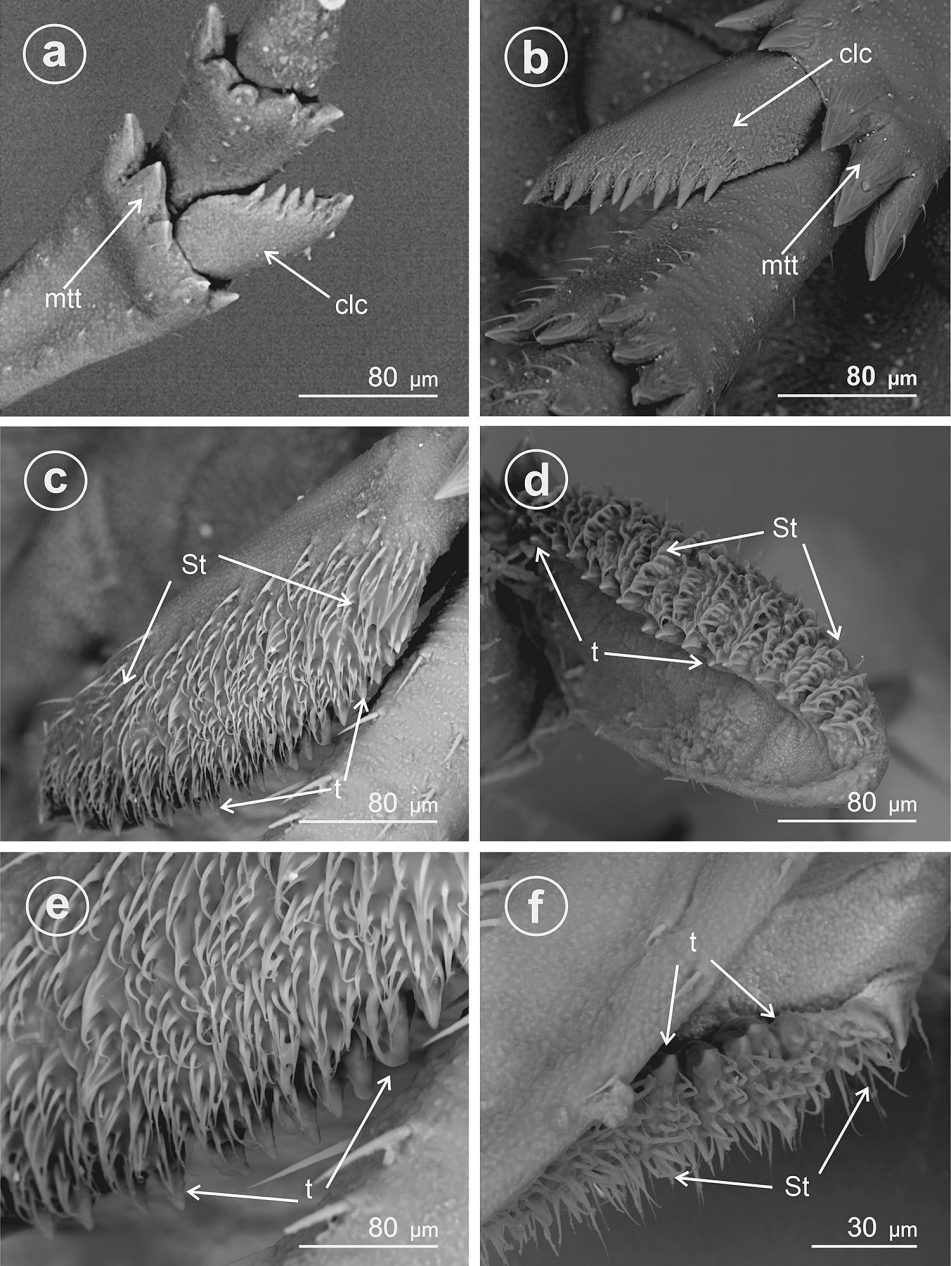
Ventral view of calcar of *Megadelphax sordidula*, (**a**) 3rd instar (**b**) 4rd instar (**c**–**f**) imago. *St* sensilla trichoidea, *clc* calcar, *mtt* metatibial apical row of teeth, *t* teeth.

In *Megadelphax sordidula* (Delphacinae: Delphacini) (Fig. 7), the 3rd instar’s calcar is short, wide, with 7 teeth; the 4th instar’s adplantar margin of calcar is more elongated and bears 9 teeth and several sensilla chaetica (Fig. 7a,b). The calcar is distinctly elongated in adults, with numerous small teeth (30) and a wide row of the sensilla trichoidea at the adplantar margin (Fig. 7c–f).

**Figure 8:**
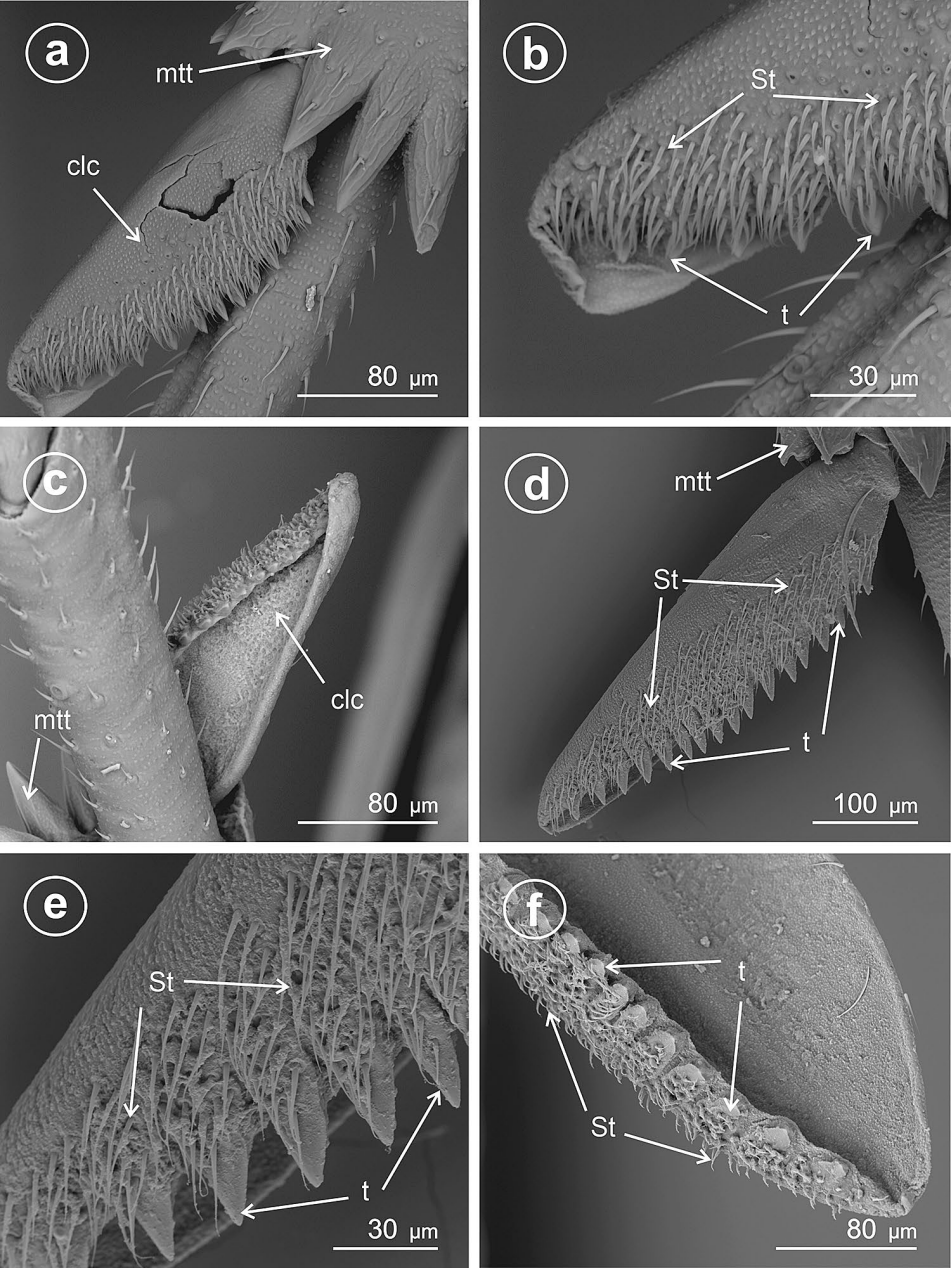
Dorsal and ventral view of calcar, (**a**,**b**,**d**,**e**) dorsal view, (**c**,**f**) ventral view. (**a**–**c**) *Xantodelphax straminea,* (**d**–**f**) *Struebingianella lugubrina*, *St* sensilla trichoidea, *clc* calcar, *mtt* metatibial apical row of teeth, *t* teeth.

In *Xanthodelphax straminea* (Fig. 8a–c) and *Struebingianella lugubrina* (Fig. 8d–f) (Delphacinae: Delphacini) the adplantar margin of calcar bears 20 teeth and abundant sensilla trichoidea.

**Figure 9:**
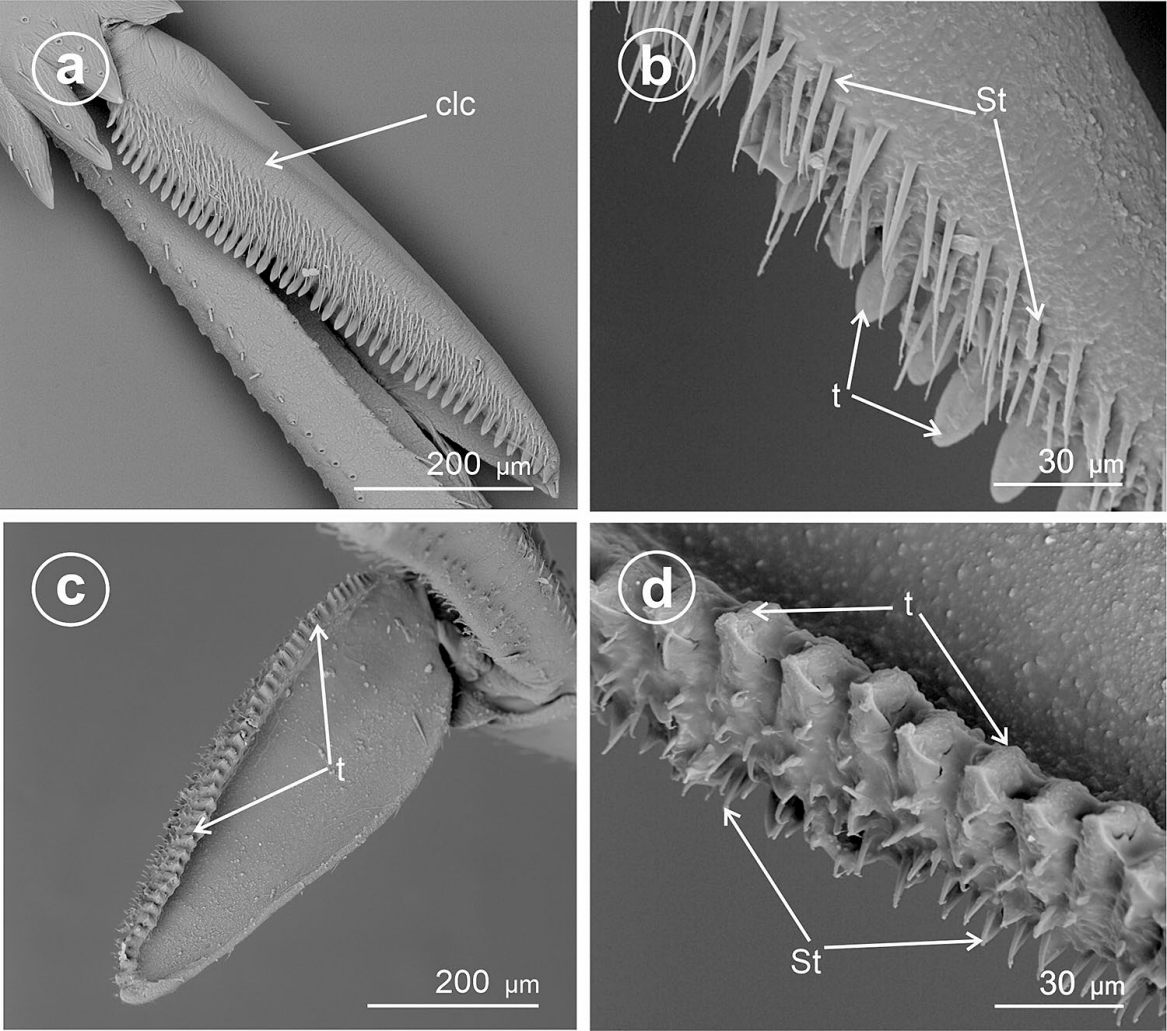
Dorsal and ventral view of calcar, (**a**,**b**) dorsal view, (**c**,**d**) ventral view of *Chloriona smaragdula*, *St* sensilla trichoidea, *clc* calcar, *t* teeth.

In *Chloriona smaragdula* (Delphacinae: Delphacini) (Fig. 9a–d), the calcar is strongly elongated, and the adplantar margin bears a row of about 40 small, densely packed teeth (t). A few rows of sensilla are located along the adplantar margin above the teeth (Fig. 9b).

### Type 8: calcar densely denticulate with bristly mechanosensilla

This type seems to be further modified than Types 4 and 5, triangular to sickle-shaped in cross-section, with the ventral adplantar margin elongated teeth, and rows of densely packed mechanosensilla and with apex diminutive.

**Figure 10:**
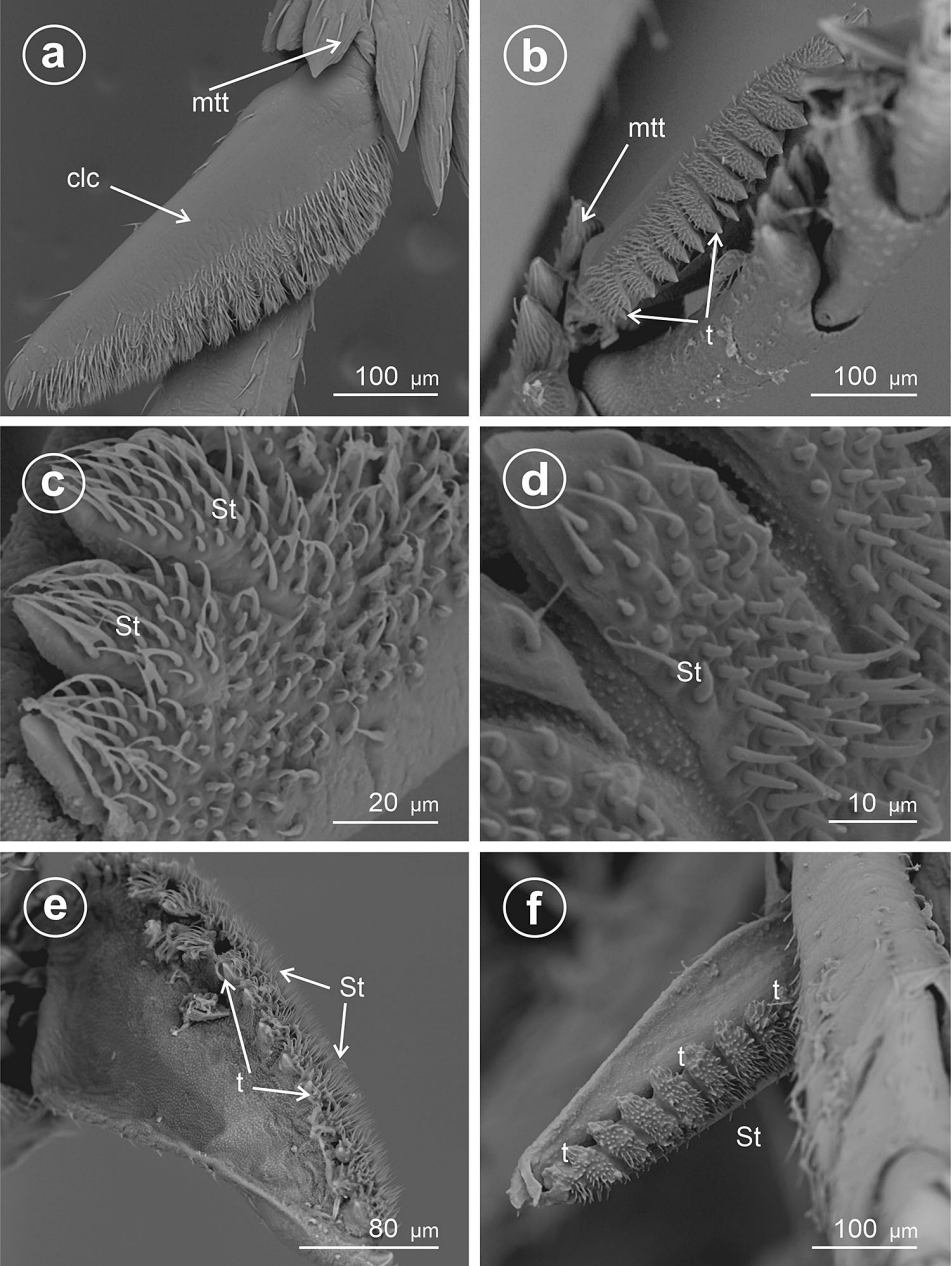
Dorsal and ventral view of calcar, (**a**–**f**) *Stenocranus fuscovittatus*, *St* sensilla trichoidea, *clc* calcar, *mtt* metatibial apical row of teeth, *t* teeth.

**Figure 11:**
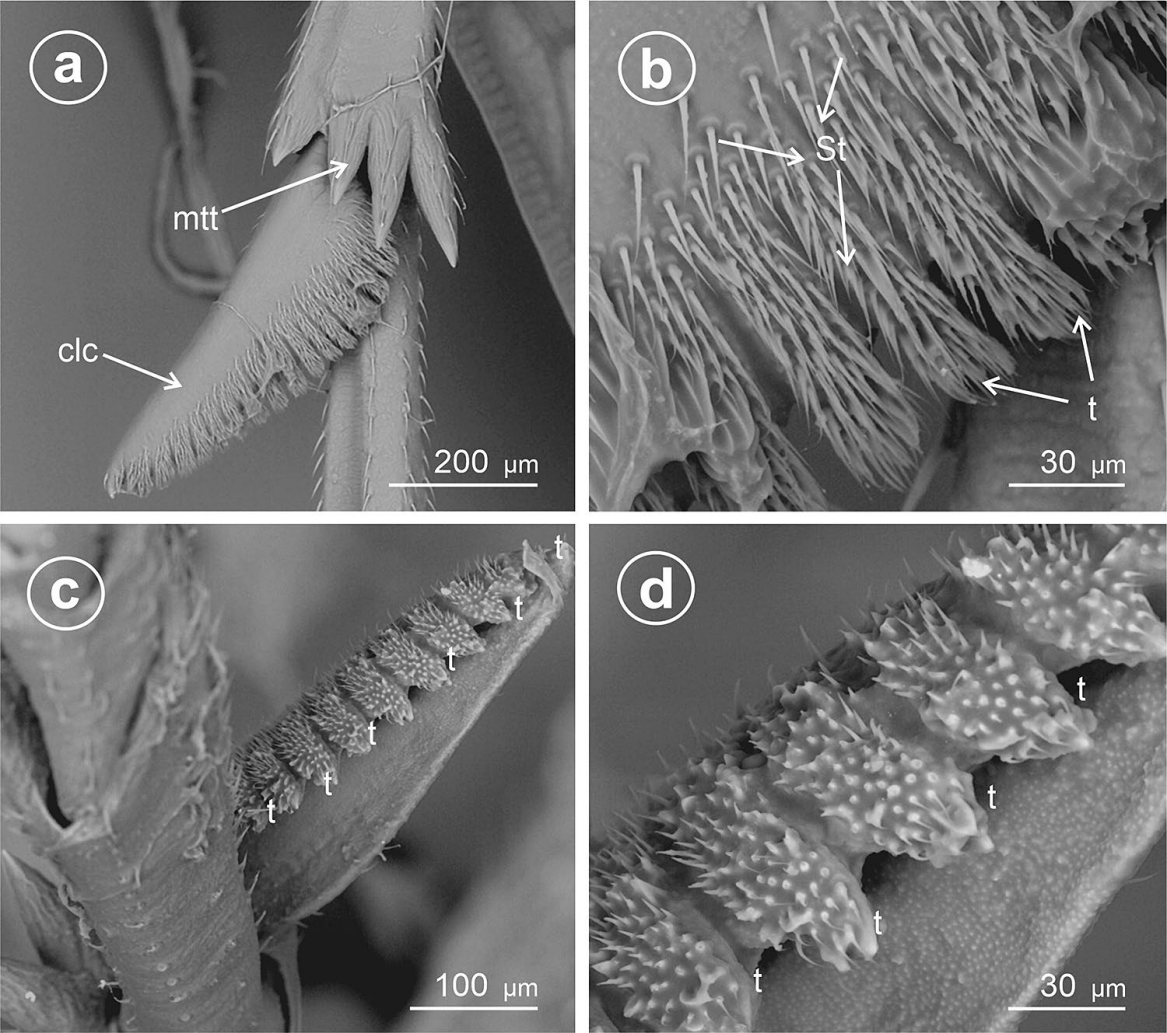
Dorsal and ventral view of calcar, (**a**–**d**) *Stenocranus major*, *St* sensilla trichoidea, *clc* calcar, *mtt* metatibial apical row of teeth, *t* teeth.

In *Stenocranus fuscovittatus* (Stenocraninae) (Fig. 10a–f), the calcar adplantar margin presents a row of stout teeth (14), but in *Stenocranus major* (Fig. 11a–d) the adplantar margin bears 20 teeth. Specific features of this type includes deep separation of each tooth on the adplantar margin and location of the brush of sensilla trichoidea along with the teeth.

### Type 9: calcar tectiform toothless (edentate)

Here the calcar is flattened, sickle-like in cross-section, but the explantar (dorsad) edge is obsolete; the edplantar edge is toothless and covered with several rows of sensilla trichoidea.

**Figure 12:**
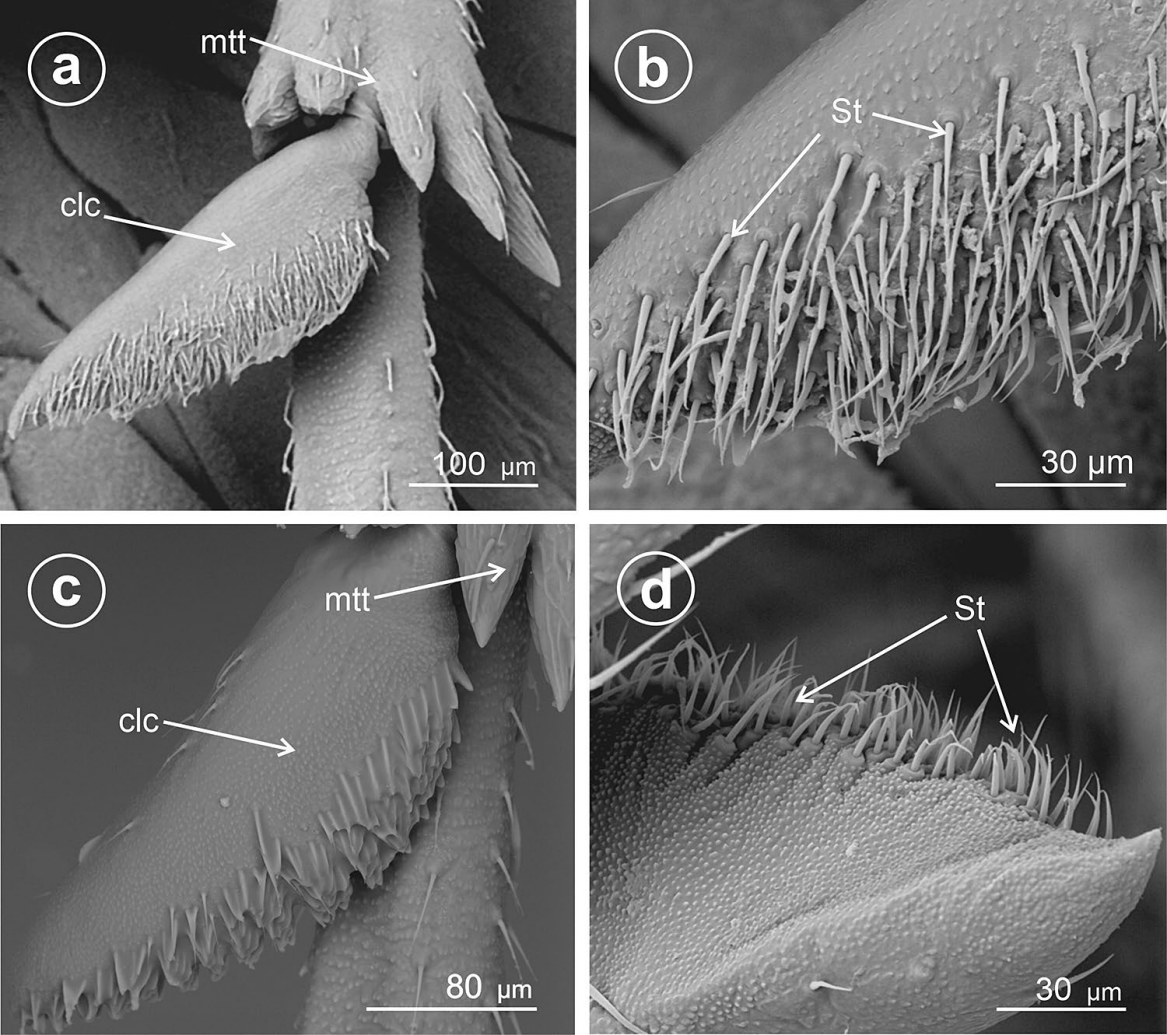
Dorsal and ventral view of calcar (**a**,**b**,**d**) *Stiroma affinis*, (**c**) *Hyledelphax elegantulus*, ventral view, *St* sensilla trichoidea, *clc* calcar, *mtt* metatibial apical row of teeth.

In *Stiroma affinis* (Fig. 12a,b,d) and *Hyledelphax elegantulus* (Fig. 12c) (Delphacinae: Delphacini) the calcar is toothless (edentate). Its edge is covered with several rows of sensilla trichoidea. The proximal and median portions ofcalcar are significantly wider than the distal, tapering portion.

## Discussion

Although a charismatic feature of Delphacidae, the calcar has not been previously the subject of detailed comparative and morpho-functional studies. The delphacid tibial calcar has been widely used in taxonomic treatments of members of this family from Muir^19^ to present times^9,68,69^. The features of calcar studied under light microscopy were presented by Metcalfe^62^, Wilson & McPherson^63,70^, Asche^27^, Liang^64^ and Bartlett & Webb^65^. The pioneering papers with the use of Scanning Electron Microscopy to study the calcar were presented by Mora et al.^71^ and Liang^64^. However, since then, no further attention has been directed to studies of calcar structure. The calcar is present also in the nymphs, since the 1^st^ instar; however it is different in size, shape and armature from those in the imagines^25,63,70,72,73^. A preliminary attempt to trace evolutionary changes of the calcar was presented by Muir^19^. Later, Wilson & McPherson^63^ and Asche^27^ discussed the features of calcar in the evolutionary aspect. A general tendency involves a change from a subulate calcar to a tectiform one (Table 1), which is variously armed and flattened.

These opinions could be confirmed by analysing the data in recent phylogenetic studies of Delphacidae^4,5^ and our results. In the present study, in SEM, the calcar differs between various tribes of Asiracinae. Type 1 calcar present in Asiracini (Asiracinae) seems to be the most plesiomorphic condition, movable, an awl-shaped and long spine, not different except size and movability from apical teeth. A slightly modified Type 1calcar, more quadrangular in cross-section is mentioned in the genera *Notuchoides* Donaldson, 1988 and *Kiambrama* Donaldson, 1988^74^. The same type of calcar is observed in other Asiracini genera *Copicerus* Swartz, 1802 and *Elaphodelphax* Fennah, 1949^27,75^.

A little more modified type, subular and with sensilla chaetica and sensilla trichoidea organized in rows (Type 2) is present in Ugyopini (Asiracinae). Within this tribe, in species of the genus *Ugyops* the calcar is elongate, subular and angulate with sensilla chaetica and sensilla trichoidea arranged in rows. Within this model variabilities are observed in particular species: a row of the sensilla chaetica and two rows of long sensilla trichoidea in *U. nemestrinus* and *U. inermis* as well as three rows of short and stout sensilla chaetica in *U. taranis*. In other known species of *Ugyops* (Table S1), the calcar presents the same model (Type 2). However, the calcar in examined *Notuchus linnavuorii* (Ugyopini) is slightly different from the species of *Ugyops* and more similar to *Asiraca* (Type 1). It was mentioned in previous works^76,77^, as short and subulate. This observation is confirmed here. Interestingly, in subterranean species of Asiracini of the genus *Notuchus* viz. *Notuchus kaori* Hoch & Asche, 2006 and *Notuchus ninguae* Hoch & Asche, 2006 the calcar is strongly diminished and vestigial^78^.

Little can be said about details of the structure of the calcar in other Asiracinae. It seems to be Type 1 in Idiosystatini, Neopunanini, Platysystatini and Tetrasteirini Emeljanov 1985^27,79–81^. In Eodelphacini, the calcar represents Type 2^82^.

**Figure 13:**
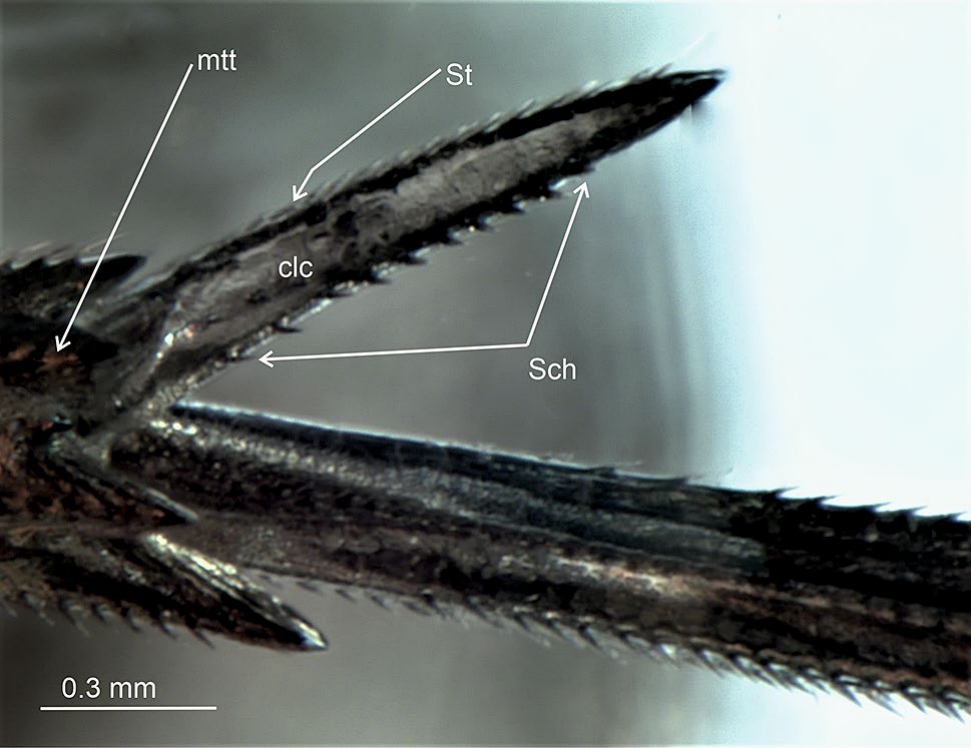
Calcar of fossil Ugyopini from Miocene Dominican amber, *St* sensilla trichoidea, *Sch* sensilla chaetica, *clc* calcar, *mtt* metatibial apical row of teeth.

A few fossil Asiracinae have been reported. In *Serafinana perperunae* Gębicki & Szwedo, 2000 from the Eocene Baltic amber (Ugyopini), the calcar is subulate, long, with setae arranged in rows as in Type 2^47^ and similar to that in Ugyopini of Dominican amber (Fig. 13). Solórzano-Kraemer^49^ briefly described another fossil from Miocene Mexican amber, placed in the genus *Eucanyra* Crawford, 1914 (synonym of *Ugyops*). The calcar in this fossil also seems to represent Type 2.

In the Vizcayinae (genera *Vizcaya* Muir, 1917 and *Neovizcaya* Liang, 2002) the calcar appears to represent Type 3—it is subulate, round in cross section with a row of teeth on the adplantar side (^28^: Figs. 35, 51,^64^: Figs. 18, 20, 31, 48, 51, 53).

A similar Type 3 calcar appears in Plesiodelphacinae: genera *Burnilia* Muir & Giffard, 1924 and *Plesiodelphax* Asche, 1985 (^83^: Figs. 284–285); the calcar is cultrate, convex on both sides and with a row of teeth on the adplantar surface^83,84^.

Type 4 calcar is present in the genera *Aloha*, *Dictyophorodelphax*, *Leialoha*, *Nesodryas*, *Nesorestias* and *Nesosydne* of Delphacinae: Delphacini (^19,27^: Figs. 294–301,^67^). Species of these genera inhabiting Hawaii and Marquesas seem to be more related to trees and shrubs of various plant families, mostly dicotyledonous, probably using a greater diversity of microhabitats available^85,86^ and having adapted to them. Asche^10^ postulated that this type of calcar evolved independently, possibly to enhance walking on particular surfaces (*e.g.*, on woody substrates). Interestingly, ‘alohine’ calcars were mentioned also in *Burnilia* (Plesiodelphacinae)^84^ but earlier Asche^83^ described the calcar in Plesiodelphacinae as “kelisioid” rather than “alohinid”. An “alohinoid” calcar was also reported for the genus *Sparnia* Stål, 1862 (Delphacini), by Asche & Emeljanov^87^.

Within the Kelisiinae (genera *Anakelisia* Wagner, 1963 and *Kelisia*), the calcar represents Type 5, with a subtriangular cross-section, a slightly concave adplantar surface and a distinct row of long, conical teeth on the adplantar margin. As observed in *Kelisia praecox* and *Anakelisia fasciata* (Kirschbaum, 1868), each tooth bears one or two mechanosensilla. The remainder of calcar surface bears sparse mechanosensilla.

In the Stenocraninae, at least these with known detailed structures of calcar, it is Type 8. This model is observed in *Stenocranus major* (Kirschbaum, 1868), *S. longipennis* (Curtis, 1837), *S. pacificus* Kirkaldy, 1907, Afrotropical *Embolophora monoceros* Stål, 1855, *Stenokelisia angusta* Ribaut, 1934, East Palearctic *Terauchiana* (*Terauchiana*) *singularis* Matsumura, 1915^27,88^. This type is also present in New World genera *Frameus* Bartlett, 2010, *Kelisicranus* Bartlett, 2006, *Obtusicranus* Bartlett, 2006, *Tanycranus* Bartlett, 2010^89–91^. It is not clearly evident from available sources, but Type 8 is probably present also in the genus *Preterkelisia* Yang, 1989^92,93^. Almost nothing is known on the calcar in the Stenocraninae genus *Proterosydne*, except it, is “… solid, elongate, narrow, with 8 spines” (^94^: 131), suggesting it could represent Type 5 or Type 8.

Within the representatives of subfamily Delphacinae the calcar diversity is the largest, with Types 5, 6, 7 and 9 distributed among various taxa. In the Saccharosydnini various types are reported, *e.g.*, various species of the genus *Saccharosydne* Kirkaldy, 1907 present Type 7 (with 18–25 teeth), *Lacertinella* Rossi Batiz et Remes Lenicov, 2012 presents Type 6 (with 14–20 teeth), *Neomalaxa* Muir, 1918 Type 6 (with 14–16 small teeth) and also in the genus *Pseudomacrocorupha* Muir, 1930 Type 6 is present (with 14 teeth)^27,30,93,95,96^. Based on the available data, representative of the tribe Tropidocephalini probably present Type 9 calcar (toothless tectiform) *e.g.*, *Malaxa* Melichar, 1914, and *Jassidaeus* Fieber, 1866 (^27^:Figs. 289–290), *Lamaxa* Bartlett & Kennedy, 2018, *Xalama* Bartlett & Kennedy, 2018^97^. According to Bartlett et al.^91^, Tropidocephalini can be recognized by the calcar being solid and triangular in cross-section and lacking teeth (although a terminal tooth is often present). This type of calcar was also reported in other genera, *e.g.*, *Tropidocephala* Stål, 1853, *Columbiana* Muir, 1919, *Jassidaeus*, *Purohita* Distant, 1906^68,69,91,98,99^.

The highest variability of calcar is known among Delphacini, *e.g*., Type 9 is present in *Paranectopia* Ding et Tian, 1981 originally described in Tropidocephalini, but moved to Delphacini^5^. Type 9 is also known in the genera *Achorotile* Fieber, 1866, *Astatometopon* Campodonico, 2017, *Eurybregma* Scott, 1875, *Hyledelphax* Vilbaste, 1968, *Nataliana* Muir, 1926, *Stiroma* Fieber, 1866^27^. Type 5 is present *e.g.*, in the genera *Conomelus* Fieber, 1866, *Onidodelphax* Yang, 1989, and species *Formodelphax formodus* Yang, 1989^93^. The calcar attributed to Type 6 is present *e.g.*, in genera *Euconomelus* Haupt, 1929, *Isodelphax* Fennah, 1963, *Javesella* Fennah, 1963, *Laodelphax* Fennah, 1963, *Megamelus* Fieber, 1866, *Muellerianella* Wagner, 1963, *Syndelphax* Fennah, 1963^100^. The calcar representing Type 7 is present in a wide spectrum of genera, *e.g*., *Delphax* Fabricius, 1798, *Delphacodes* Fieber, 1866, *Ditropis* Kirschbaum, 1868, *Abbrosoga* Caldwell, 1951, *Bostaera* Ball, 1902, *Chloriona* Fieber, 1866, *Lepidelphax* Lenicov & Walsh, 2013, *Megadelphax* Wagner, 1963, *Megamelodes* LeQuesne, 1960, *Nilaparvata* Distant, 1906, *Nycheuma* Fennah, 1964, *Pseudaraeopus* Kirkaldy, 1904, *Sogatella* Fennah, 1956, *Struebingianella* Wagner, 1963, *Thymalops* Fennah, 1965, *Unkanodes* Fennah, 1956, *Xanthodelphax* Wagner, 1963^27,69,101,102^.

The exact function of calcar and its mode in Delphacidae remains unresolved. The reasons for the disparity of calcar structures are not well recognized. It is generally believed that the calcar is used to assist in jumping; however, observations and experiments have not confirmed it. The recent observations of Delphacidae jumping mechanisms focused on the thorax, its musculature and base of legs with no attention to the role or function of calcar at the process^103,104^. With the presence of several types of sensory hairs, the calcar seems to be involved in the process of jumping, but its variability could be related to the surfaces from which the jump is taken. It seems plausible that this diversity is somewhat related to the structure and properties of the surface on which planthopper is living. Delphacids are relatively host-specific, and most mainland species (92% of records) attack monocots; dicot feeding dominates (82% of records) only on oceanic islands^3,10,67^. The Asiracinae Ugyopini recorded on dicotyledones mainly feed on woody dicots (most probably secondarily) and on monocotyledon Arecaceae. The family Arecaceae comprises 240 genera and approximately 2700 species predominantly concentrated in tropical and subtropical regions^105,106^, with fossil record reaching Late Cretaceous^107^. Type 2 calcar, as in Ugyopini, could be, on the one hand, a conservative model and, on the other, an expression of adaptation and a long co-evolutionary history with their host plants. Type 1 calcar, is present in Asiracini; for these planthoppers, most monocot records is related to Cyperaceae, but some are recorded on dicots and even ferns (this is a definitively secondary adaptation to host plant). Cyperaceae crown groups appeared in the Late Cretaceous-Early Paleogene, but their diversification took place at the end of the Eocene^108,109^. Host plants of Vizcayinae remain unknown; Kelisiinae seem to be strictly associated with Cyperacae and Juncaceae, plants of extraordinary ecological importance, occupying a broad range of habitats from rain forests to tundra, as components of open habitats including many types of wetlands, temperate and tropical grasslands and savannas, especially in moist sites, or more shaded ones as understory of forests^110,111^. The evolutionary shifts in the history of these plants took place at the terminal Eocene-Early Oligocene, during a global cooling period and in the Miocene, during global warming, then cooling periods, resulting in rapid diversification^111,112^. Therefore it could be assumed that Kelisiinae retaining Type 5 of calcar shifted to these host plants simultaneously with or after their diversification and spreading. In Stenocraninae, the calcar of Type 8 is present, which could be related to adaptations to a broader array of host plants. Stenocraninae seem to be strongly associated with monocotyledons in modern fauna, with a clear dominance of Poaceae over Cyperaceae^3,90^. In Delphacinae, the variability of types of calcar is the highest (Table1); while in vast majority they feed on various Poaceae, shifts to other monocots or dicotyledons are known^3,4^. Also, modifications of calcar were reported in Delphacini taxa associated with water plants (*e.g*., waterlily), *e.g*. *Megamelus davisi* Van Duzee, 1897 with an exceptionally large, thin and leaflike calcar with about 20 small teeth, but allowing to place it to Type 7^113–116^. The shape of the calcar probably facilitates jumping from the water surface (personal communication, Ch. Bartlett). Delphacini seem to have experienced several host shifts but do not present a co-evolutionary pattern; representatives of this lineage are clearly ecological opportunists^4^. Therefore the variability and disparity of calcar and its pattern in this group is homeoplasous, not giving strong phylogenetic signal, but could be a good tool to understand the ecological history of the Delphacini and their temporal shifts and adaptations to host plants. Increased diversification within Delphacini may reflect a shift to grass-feeding, and host shifts within Poaceae, perhaps from grasses with C3–C4 photosynthetic pathways^4^, and different anatomical features of C3 and C4 grasses^117^. It must be noted that host plant associated diversification within Delphacidae was mediated by co-evolutionary relationships with endosymbiotic bacteria and fungi^118,119^; therefore, the natural selection and adaptation of these planthoppers took place at various planes.

The calcar is the most significant character defining Delphacidae but its evolutionary origin is not fully resolved. Basing on its postembryonic development, it is supposed that the calcar is derived from one of lateral apical teeth of the hind-tibia as present in Cixiidae^29,63,120^. Modern Cixiidae, as well as most of the known fossils, present some variability in the armature of metatibial apex; however, the elongate outer tooth of the external group often stands out, and the two next teeth of the same group are shorter; these three teeth form a medial group, and three external teeth form an opposed group. In the tribes Oecleini Muir, 1922 and Gelastocephalini Emeljanov, 2000 the teeth of external and medial groups are separated with an interspace—the row is interrupted by diastema^121^. Such a pattern could correspond to the condition present in Delphacidae ancestors. Emeljanov^29^ discussed the possible transformations of metatibial apical teeth proposed earlier by Asche^27^ and interpreted formation of calcar setation differently; he postulated four indistinct rows of setae as a plesiomorphic condition with further transformations due to metatopy. Recently Fu et al.^122^ indicated that the cloned full-length *Ubx* ortholog (*NlUbx*) activates the development of spines on the T3 tibia and basitarsus. The Hox gene Ultrabithorax (*Ubx*) plays pivotal roles in modifying specific morphological differences between T2 and T3 in various hemipterans^122–124^. However, more data and observations are needed to fully understand the developmental and evolutionary ways of calcar origination and formation.

The fossil record of Delphacidae is very scarce; Eocene Baltic amber inclusion *Serafinana perperunae* Gębicki & Szwedo, 2000, represents Ugyopini, Solórzano Kramer (2007) mentioned not formally described Ugyopini (identified as *Eucanyra*—synonym of *Ugyops* Guérin-Méneville, 1834) from Miocene Mexican amber; more Ugyopini were found as inclusions in Miocene Dominican amber (Fig. 13). The other Miocene taxon: *Amagua fortis* Cockerell, 1924 from Late Eocene/Early Oligocene of Amgu (Amagu) River, Sikhote-Alin, Russia is a fragmentary imprint of forewing and body, not to be placed in any subfamily or tribe; *Chloriona stavropolitana* Becker-Migdisova, 1964 from the Messinian, Miocene of Vishnevaya Balka, Northern Caucasus, Russia based on partly preserved tegmen, seems to be the oldest record of Delphacini. It is doubtful whether the Ypresian, Eocene, and Green River Formation fossils: *Delphax senilis* Scudder, 1870 and *Delphax veterum* Cockerell, 1921 should be assigned to Delphacidae and the original materials must be revised; Chattian, Oligocene fossil named ‘*Delphax*’ *rhenana* Statz, 1950 does not seem to represent Delphacidae^48,50,52–54^. The calcar from fossil resins is not different from the one present in modern Ugyopini. The taphonomic potential of Delphacidae for fossilisation as adpressions seems not to be high due to various extrinsic and intrinsic factors^125,126^; therefore, the inclusions in amber and other fossil resins remain an invaluable source of information.

## Conclusion

The calcar of Delphacidae is their unique synapomorphy, defining the family as a whole. Its structure is highly variable, but the use and estimation of its phylogenetic and classification values seem challenging, as detailed knowledge of theof calcar structure and its function remains limited. On the other hand, the variability and disparity of calcar and its patterns observed in Delphacidae could be a good tool to understand the ecological history of these insects and their temporal shifts and adaptations to host plants. Being a charismatic character of the Delphacidae, the calcar still poses a number of problems to be addressed, *e.g.*, its evolutionary origin, the developmental ways of formation, factors influencing its disparity, even the exact function. Here, we presented the first attempt to systematize calcar models and structures and evaluate its potential in morphological, evolutionary and ecological studies. We have also justified the need for restudying this morphological structure.

## Supplementary Information

Supplementary Information.

## Individual

USMB: Upper Silesian Museum in Bytom
DZUS: Department of Zoology, University of Silesia
LTIB: Laboratory of Terrestrial Invertebrates: State Scientific and Production Amalgamation The Scientific and Practical Center for Bioresources, National Academy of Sciences of Belarus

## Morphological

mtt: Metatibial apical row of teeth
ms: Membranous connection of the calcar
clc: Calcar
s: Socket
m: Flexible membrane
t: Calcar tooth
St: Sensillum trichoideum
Sch: Sensillum chaeticum
