# Supplementary material for: Morphological reassessment of the movable calcar of delphacid planthoppers (Hemiptera: Fulgoromorpha: Delphacidae)
Source: Sci Rep. 2021 Nov 16;11:22294. doi: 10.1038/s41598-021-01771-9 (PMC8595309; doi:10.1038/s41598-021-01771-9)
Supplement: Supplementary file 1 — Supplementary Information. [file 41598_2021_1771_MOESM1_ESM.pdf]

Running title: Movable calcar of delphacid planthoppers

Morphological reassessment of the movable calcar of delphacid planthoppers (Hemiptera: Fulgoromorpha: Delphacidae)

Darya Markevich [1], Marcin Walczak [2], Oleg Borodin [3], Jacek Szwedo [4]*, Jolanta Brożek [2]*

[1] National Academy of Sciences of Belarus, State Scientific and Production Amalgamation The Scientific and Practical Center for Bioresources, Laboratory of Terrestrial Invertebrates, 27, Akademicheskaya Str., 220050 Minsk, Belarus

[2] Faculty of Natural Sciences, Institute of Biology, Biotechnology and Environmental Protection, University of Silesia in Katowice, 9, Bankowa St., PL40-007 Katowice, Poland

[3] Daugavpils University, Institute of Systematic Biology, 13 – 229 Vienības Street, Daugavpils LV-5401, Latvia

[4] Laboratory of Evolutionary Entomology and Museum of Amber Inclusions, Department of Invertebrate Zoology and Parasitology, Faculty of Biology, University of Gdańsk, 59, Wita Stwosza St., PL80-309 Gdańsk, Poland

*Corresponding Authors

jacek.szwedo@biol.ug.edu.pl; jolanta.brozek@us.edu.pl

Supplementary Table S1

Table S1. Review of previous authors' information on calcar in many delphacid species based on a light microscope. Terminology: calcar (post-tibila spur, metatibial spur), teeth =spines, hair- mechanosensilla.

Subfamily/ tribe/genus	Species	Calcar description	Authors of publication
Asiracinae Motschulsky, 1863	calcar is spinelike (not flattened) and lacks teeth, calcar circular or quadrangular in cross section		https://sites.udel.edu/ planthoppers/north-america/north-american-delphacidae /subfamily-asiracinae -fieber-1872/[127], Asche (1990)[28]
	Pentagramma bivittata Crawford, 1914<br>*Pentagramma cosquina* Penner, 1947<br>*Pentagramma nimbata* (Berg, 1879)<br>*Pentagramma vittatifrons* (Uhler, 1876)	calcar rather long, terete, spiniform acute at tip	https://sites.udel.edu/ planthoppers/north-america/north-american-delphacidae/genus-pentagramma-van-duzee-1897/[127]

Idiosystatini Emeljanov, 1995	*Idiosemus xiphias* (Berg, 1879)<br>*Idiosystatus fuscoirroratus* (Spinola, 1852)<br>*Idiosystatus acutiusculus* (Spinola, 1852)<br>*Idiosystatus australis* Fennah, 1969<br>*Idiosystatus gubernator* Fennah, 1969<br>*Idiosystatus longifrons* Fennah, 1969	calcar cultrate (sharp-edged and pointed) with short hair	https://sites.udel.edu/ planthoppers/north-america/north-american-delphacidae/tribe-idiosystanini-Emeljanov-1995/[127]
Ugyopini Fennah, 1979	*Ugyops godmani* (Fowler, 1905)	calcar long, triangular-prismatic, faces flat, acute at tip, finely pubescent	Asche (1985b)[27], https://sites.udel.edu/ planthoppers/north-america/north-american-delphacidae/tribe-ugyopini-fennah-1979/[127]
	Ugyops stigmatus (Crawford, 1914)	calcar very long, slender, triangular-prismatic, slightly pubescent, with a row of teeth= bristles posteriorly, acute tip	
	Ugyops supercilliatus Fennah, 1956<br>*Ugyops kelleris* Muir, 1921<br>*Ugyops zimmermani* Fennah, 1950<br>*Melanugyops erebea* Fennah, 1956	calcar very long	
	Ugyops bifurcatus (Metcalf, 1945)	calcar quadrate	Caldwell & Martorell (1951)[101]
	Ugyops brunneus (Fowler, 1905)	calcar quadrate	
	Notuchus larvalis Fennah, 1980	calcar short, stout	Fennah (1980)[128]
	Notuchus risioides Fennah, 1969<br>*Notuchus monticola* Fennah, 1973<br>*Notuchus rotundifacies* Donaldson, 1979<br>*Notuchus kurandae* Donaldson, 1979	calcar subulate, moderately long	Donaldson (1979)[76]
	Notuchus kaori Hoch & Asche, 1986	calcar minute	Hoch et al. (2006)[78]
	Notuchus ninguae Hoch & Asche, 1986	calcar vestigial	
AsiraciniFieber, 1872	*Copicerus irroratus* Swartz, 1802	calcar spine-like (not flattened) lacking teeth along the posterior margin,<br>calcar long, very acute at tip, slightly pubescent	Asche (1985b)[27], 1990)[28], Emeljanov 1995)[29], Crawford (1914)[129]
	Asiraca clavicornis (Fabricius, 1794)<br>*Asiraca germari* Metcalf, 1943	calcar is spiniform	Asche (1985b)[27], sites.udel.edu/planthoppers/north-america/north-american-delphacidae/subfamily-asiracinae-fieber/[127]
	Copicerus insignicornis (Lethierry, 1890)<br>*Copicerus obscurus* (Guerin-Meneville, 1856)<br>*Copicerus swartzi* Stål, 1857<br>*Elaphodelphax nigropicta* Fennah, 1949	calcar long, very acute at tip, slightly pubescent, without teeth	
Neopunanini Emeljanov, 1995	*Neopunanapuertoricensis* Muir, 1918<br>*Neopunana vulgaris* (Caldwell, 1951)<br>*Neopunana saba* Asche 1983<br>*Neopunana alapa* (Caldwell, 1951)<br>*Neopunana caribbensis* (Caldwell, 1951)	calcar is spiniform	https://sites.udel.edu/ planthoppers/north-america/north-american-delphacidae / tribe-neopunanini-emeljanov-1995-and-genus- neopunana -Asche-1983/[127]
Eodelphacini Emeljanov, 1995	*Melanesia pacifica* Kirkaldy, 1907	calcar quadrate short	https://sites.udel.edu/ planthoppers/north-america/north-american-delphacidae / tribe-ugyopini-fennah-1979/[127]
	Punana annulata (Distant, 1916)	calcar is spiniform	
Platysystatini Emeljanov, 1995	*Platysystatus brunneus* Muir, 1930<br>*Platysystatus itapetingus* Asche, 1983	calcar is spiniform, relatively short	https://sites.udel.edu/ planthoppers/north-

	Pentasteira albifrons Baringer&Bartlett, 2011<br>*Pichinchana gilletti* Asche and Webb, 2013<br>*Equasystatus breviceps (*Muir, 1926)		america/north-american-delphacidae/tribe-platysystatini-emeljanov-1995/[127]
Tetrasteirini Emeljanov, 1996	*Tetrasteira albitarsis* Fennah, 1945<br>*Tetrasteira minuta* Muir, 1926<br>*Tetrasteira solata* Barringer & Bartlett, 2011<br>*Tetrasteira trimaculata* Barringer & Bartlett, 2011<br>*Tetrasteira vulgaris* Barringer & Bartlett, 2011	calcar is spiniform,	https://sites.udel.edu/planthoppers/north-america/north-american-delphacidae/tribe-tetrasteirini-emeljanov-1995-and-genus-tetrasteira-muir-1926/[127]
Plesiodelphacinae Asche, 1985	calcar circular in cross section, with distinct conical teeth		Asche (1990)[28]
Plesiodelphacini Asche, 1985a	*Burnilia pictifrons* (Stål, 1864)	calcar half as long as elongate basal tarsus, coarsely 8-dentate, acute at tip	Crawford (1914)[129]
	Burnilia japonica Asche, Hayashi &Fujinuma, 2016	calcar “alohine”, i.e., elliptical in cross-section bearing well separated cone-shaped teeth at the posterior margin~~;~~	Asche et al. (2016)[84]
	Plesiodelphax guayanus Asche, 1985	calcar long, several teeth evidently separated and with singular setae	Asche (1985a)[27]
Vizcayinae Asche, 1990	calcar with solid conical teeth on inner margin		Asche (1990)[28]
	Neovizcaya sinica Liang, 2002	calcar with 5teethon inner margin	Liang (2002)[64]
	Vizcaya bakeri Muir, 1917	calcar assymetricallly dentated, with six (left) + eight (right) teeth	Asche (1990)[28]
	V. orea Asche, 1990	calcar with 9-11 teeth	
	V. adornata Asche, 1990	calcar with 7 teeth	
	V.piccola Asche, 1990	calcar with 6 teeth	
	Vizcaya vindaloa Asche, 1990	calcar with 9 (left) and 12 (right) teeth	
	V. aschei Liang, 2002	calcar with 9 teeth	Liang (2002)[64]
	V. latifrons Liang, 2002	calcar with 7 teeth	
	V. longispinosa Liang, 2002	calcar with 8±11 teeth (number of teeth varying among individuals and on left and right legs)	
	V. lombokensis Liang, 2002	calcar - 6 teeth	
Delphacinae Leach, 1815	calcar variously shaped, solid or flattened, normally with teeth on inner margin		Asche (1990)[28]
Delphacini Leach, 1815	*Ambarvalia pyrops* Distant, 1917	calcar unusually small, with 3-6 teeth	Fennah (1964)[130]
	Nesodryas antiope (Fennah, 1964)	calcar with 6-12 teeth	
	Nesodryas antiope seychellensis Fennah, 1964	calcar with about 7 teeth	
	Rhinodelphax ion Fennah, 1964	calcar with 17 teeth	
	Thymobares ismenis Fennah, 1958	calcar moderately thin with 11-22 teeth	
	Thymobares longispinus Muir, 1929	calcar moderately thin with 11-22 teeth	

	Oaristes snelli (Muir, 1929)	calcar with about 21 teeth	
	*Tagosodes elpeno*r (Fennah, 1964)	calcar with 18 teeth	
	Thriambus stramineus (Muir, 1929)	calcar is short and bears less than 15 very minute teeth	
	Nycheuma anderida (Kirkaldy, 1907)	calcar with15–22 teeth calcar thin and large	
	Tagosodes cubanus (Crawford, 1914)	calcar long, large, thin, margin finely dentate	
	Pissonotus marginatus Van Duzee, 1897	calcar half as long as basal tarsusfinely dentate	
	Nycheuma endymion (Fennah, 1958)	calcar thin, moderately large, with about 20 teeth	
	Numatodes antricauda Fennah, 1964	calcar with 32 teeth	
	Cemus leviculus Fennah, 1964	calcar with 30 teeth	
	Nycheuma cognatum (Muir, 1917)	calcar with about 27 teeth	Hou X-H and Chen X-S (2014)[131]
	Nycheuma dimorpha (Matsumura, 1910)	calcar with about 26 teeth	
	Nycheuma nilotica Linnavuori, 1973	calcar with about 26 teeth	
	Peregrinus maidis (Ashmead, 1890)	calcar narrow, almost triangular cross section with base concave, teeth very minute.<br>Calcar about half as long as basal tarsus	Caldwell & Martorell (1951)[101], Crawford (1914)[129]
	Gelastodelphax histrionicus Kirkaldy, 1906	calcar thin tectiform with 1 row of about 35 teeth and with apical spine	Bellis & Donaldson (2016)[132]
	Sardia rostrata Melichar, 1903	calcar with about 20 teeth	
	Syndelphaxdisonymos (Kirkaldy, 1907)	calcar with 16–24 (average 19) teeth	
	Delphax crassicornis (Panzer, 1796)	calcar very large, tectiform, deeply concave on one surface, margin finely dentate.	
	Terthron anemonias (Kirkaldy, 1907)	calcar with about 20 teeth	
	Nilaparvata albotristriata (Kirkaldy, 1907)	calcar flattened with 2 rows of teeth, 15–18 in total and with apical spine	
	Queenslandicesa fennahi Bellis & Donaldson, 2016	calcar with 15–22 teeth	
	Yangdelphax jihyuetanica Bellis & Donaldson, 2016	calcar with 32–36 teeth	
	Abbrosoga errata Caldwell, 1951	calcar large (tectiform and smooth, bearing 24–32 fine teeth on trailing edge,	Otero & Bartlett (2019)[133]
	Abbrosoga multispinosa Otero & Bartlett, 2019	calcar tectiform and smooth, bearing 27–30 fine teeth on trailing edge	
	Lepidelphax pistiae Remes Lenicov & Walsh, 2013	calcar, slender and foliaceous, as long as basitarsus, bearing 20 to 22 regular conical teeth including the distal one on outer margin; outer margin slightly convex.	Remes Lenicov & Walsh (2013)[114]
	Megamelus bellicus Remes Lenicov & Sosa, 2007	calcar more indented, reaching 15-20 marginal	Sosa & Remes Lenicov (2007)[116]

		regular-sized teeth in adults and in general aspects they are longer	
	Megamelus scutellaris Berg, 1883	calcar reaching 13-14 marginal irregular-sized teeth in adults.	
	Megamelus davisi Van Duzee, 1897	calcar laminate with several spines	Wilson & McPherson (1981b)[63]
	Megamelus bicolor Ball, 1902	calcar short, thick, and less foliaceous finely pubescent	Crawford (1914)[129]
	Prokelisia salina (Ball, 1902)	calcar thick, finely pubescent, scarcely more than half as long as basal tarsus	
	Prokelisia crocea (Van Duzee, 1897)	calcar typical, rather large, finely dentate	
	Tarophagus proserpina (Kirkaldy, 1907)<br>*Tarophagus colocasiae* (Matsumura, 1932)	calcar convex, both sides with row of 7-11 teeth	Wilson & Tsai (1988)[134]
	Javesella pellucida (Fabricius, 1794)	calcar large, often nearly as long as basal tarsus, margin distinctly dentate	Crawford (1914)[129]
	Nothodelphax consimilis (Van Duzee, 1897)	calcar short, less distinctly dentate	
	Megamelus gillettei (Van Duzee, 1897)	calcar a little more than half as long as basal tarsus, marginal dentation scarcely visible	
	Megamelus rotundatus Crawford, 1914	calcar rather thin, large, margin distinctly dentate.	
	Megamelus palaetus (Van Duzee, 1897)	calcar tectiform, the margins usually rather close together with pubescence, unusually large and foliaceous	
	Megamelus notulus (Germar, 1830)	calcar is large more than half as long as basal tarsus with numerous sharp teeth	
	Megamelus iphigeniae Muir, 1926	calcar long flattened with numerous sharp teeth	
	Muirodelphax arvensis (Fitch, 1851)	calcar thin, more than half as long as basal tarsus, margin very minutely dentate.	
	Pareuidella magnistylus (Crawford, 1914)	calcar large, broad, as long as basal tarsus, margin with numerous large teeth	
	Kormus artemisiae Fieber, 1866	calcar thick, tectiform, margin scarcely dentate. Related to Megamelus at the " Kelisia" end of the series	
	Kormus californicus Crawford,1914	calcar pubescent, more than half as long as basal tarsus,margin scarcely dentate.	
	Phyllodinus nervatus Van Duzee, 1897	calcar tectiform, margin coarsely or finely dentate, thick, stout, not very long,	
	Liburniella ornata (Stål, 1862)	calcar small, margin scarcely dentate	

	Laccocera vittipennis Van Duzee, 1897	calcar thick at base, not very large	
	Achorotile albosignata (Dahlbom, 1850)	calcar foliaceous, stout; about half as long as basal tarsus, stout, briefly pubescent	
	Bostaera nasuta Ball, 1902	calcar large and broad, three-sided, each side flat, not foliaceous; there are a number of very minute spines, scarcely visible, on one edge	
	Stobaera tricarinata (Say, 1825)	calcar cultrate (sharp-edged and pointed), with a row of 6 to 10 teeth on hind margin. long; from one-half to two-thirds as long as basal tarsus	
	Stobaera pallida Osborn, 1905	calcar more than half as long as basal tarsus	
	Bakerella maculata Crawford, 1914	calcar very short, thick, margin scarcely dentate	
	Delphax crassicornis (Panzer, 1796)	calcar large, tectiform, deeply concave on one surface, margin finely dentate	
	Nilaparvata myersi Muir, 1923	calcar with 20 teeth	Planthopper: Threat to Rice Production in Asia[135]
	Nilaparvata lugens (Stål, 1854)	calcar elongated with about 30-36 teeth	
	Sogatella unidentata Mariani & Remes Lenicov, 2017	calcar leaf-like, bearing 23–25 regular teeth on trailing margin	Mariani & Remes Lenicov (2018)[136]
	Tagosodes orizicolus (Muir, 1926)	Calcar, has on its internal edge with serrated structures containing numerous setae forming disorganized rows	Mora, Retana & Espinoza (2001)[71]
	Spartidelphax penedetectus (Beamer, 1950)	calcar with 18–31 teeth	Bartlett & Webb (2014)[65]
	Spartidelphax detectus (Van Duzee, 1897)<br>*S. luteivittus* (Walker, 1851)	calcar with 25 teeth	
	Ampliphax grandis Bartlett & Kunz, 2015	calcar large, flattened and foliaceous, narrowed distally, row of more than 28–35 teeth on posterior lateral margin	Barlett & Kunz (2015)[137]
	Syntperon brazilensis Muir, 1926	the presence of a “normally” dentate (not “alohinoid”) calcar”	Asche & Emeljanov (2016)[87]
	Leialoha mauiensis Muir, 1919<br>*Nesodryas freycinetiae* Kirkaldy, 1908<br>*Nesosydne sappho* Fennah, 1955<br>*Nesorthia paronychiae* Fennah, 1962<br>*Nesorestias filicicola* Kirkaldy, 1908<br>*Ilburnia koae* (Kirkaldy, 1907)<br>*Ilburnia ignobilis* (White, 1878)<br>*Sparnia praecellens* Stål, 1862<br>*Dictyophorodelphax mirabilis* Swezey, 1907	calcar is elongated, narrow and the one margin is toothed 7-9 stout teeth, no setae on teeth, the surface is covered by several setae	Asche (1985b)[27]
	Stobaera pallida Osborn, 1905	calcar is short and possess a 3-6 teeth	Asche (1985b)[27]

	Ambarvalia pyrops Distant, 1917	calcar with three teeth	
Delphacini Leach, 1815	*Conomelus anceps* (Germar, 1821)	calcar with teeth (9)	
	Eurybregma nigrolineata Scott, 1875	calcar tectiform, small teeth, the margin with teeth is covered densely by setae	
	Stiroma affinis Fieber, 1866	calcar tectiform, triangular no teeth, the margin covered densely by setae	
	Dicranotropis (*Leimonodite*) *divergens* Kirschbaum, 1868	calcar tectiform, triangular no teeth, the margin covered rarely by setae	
	Pseudaraeopus lethierryi (Mulsant & Rey, 1879)	calcar tectiform, triangular, very small teeth, the margin with teeth is covered rarely by setae	
	Delphacodes venosus (Germar, 1830)	calcar tectiform, oval, very small teeth about 20, the margin with teeth is covered rarely by setae	
	Ditropis pteridis (Spinola, 1839)	calcar tectiform, the margin with teeth is covered rarely by setae	
	Megamelodes quadrimaculatus (Signoret, 1865)	calcar tectiform, oval, very small teeth about 12, the margin with teeth is covered rarely by setae	
	Eumetopina hancocki Muir, 1929<br>*Nataliana lineata* Muir, 1926<br>*Calligypona reyi* (Fieber, 1866)	calcar elongate oval with point end, teeth different (smaller and longer) the egde with teeth is covered densely by setae	
	Spinidelphacella hargreavesi (Muir, 1929)	calcar tectiform, small with several short teeth, setae rarely arrangement	
	Araeopus pulchellus (Curtis, 1833)	calcar tectiform, elongate oval with point end, teeth small, the margin with teeth is covered densely by setae	
	Rhinodelphax hargreavesi Muir, 1934	calcar elongate oval with point end, teeth long, the margin with teeth is covered densely by setae	
	Chloriona vasconica Ribaut, 1934		
	Astatometopon sakakibarai Campodonico, 2017	calcar without teeth on its hind margin	Campodonico (2017)[138]
Saccharosydnini Vilbaste, 1968	*Saccharosydne saccharivora* (Westwood, 1833)	calcar spines 13-21, usually 15-19 teeth, (16s pecimens out of 20)	Metcalfe (1969[1968])[62]
	Saccharosydne rostifrons (Crawford, 1914)	calcar typical, pubescence slight, evidently tooted	Crawford (1914)[129]
	Neomalaxa flava Muir, 1918	calcar pubescence slight, several teeth	Caldwell & Martorell (1951)[101]
	Pseudomacrocorupha wagneri Muir, 1930	calcar pubescence slight, several teeth	
Tropidocephalini Muir, 1915	*Tropidocephala tuberipennis* (Mulsant & Rey, 1855)<br>*Purohita fuscovenosa* Muir, 1913<br>*Jassidaeus lugubris* (Signoret, 1865)	calcar is more oval and wider not teethed and covered with many short setae	Asche (1985b)[27]
	Malaxa microstyla Muir, 1930	calcar is narrowed in the end, triangular with many setae	

Stenocraninae Wagner, 1963	calcar foliate, tectiform, with numerous small teeth on the posterior margin,<br>calcar dilated, flattened, numerous small teeth with rectrangular platelike on inner margin		Bartlett, O'Brien &Wilson (2014)[9], Asche (1990)[28]
	Stenocranus minutus (Fabricius, 1787*)*	calcar tectiform, the margins usually rather close together with pubescence	Crawford (1914)[129]
	Stenocranus dorsalis (Fitch, 1851)	calcar large, half as long as basal tarsus, pubescent	
	Stenocranus angustus Crawford, 1914	calcar slender	
	Stenocranus similis Crawford, 1914	calcar large	
	Stenocranus longipennis (Curtis, 1837)	tectiform with a row of about 16 teeth	
	Proterosydne arborea Kirkaldy, 1907	calcar subterete, cultrate, with a row of about eight teeth on hind margin	
	Stenokelisia angusta Ribaut, 1934	calcar subterete, cultrate, with a row of about 16 teeth on hind margin	Asche (1985b)[27]
	Embolophora monoceros Stål, 1855	calcar large about 25 teeth, covered by setae	
	Terauchiana singularis Matsumura, 1915	calcar large about 25 teeth, covered by setae	
	Stenocranus pacificus Kirkaldy, 1907	calcar large with 24-25 or 26-27 teeth	Susilo et al. (2017)[88]
Kelisiinae Wagner, 1963	calcar solid, slightly concave on inner surface, with large distinct onical teeth on posterior margin		Asche (1990)[28]
	Anakelisia fasciata (Kirschbaum, 1868)<br>*Kelisia irregulata* Haupt, 1935<br>*Kelisia monoceros* Ribaut, 1934	calcar tetciform, is about 7-13 teeth with with two hairs on the long teeth	Asche (1985b)[27]

*References are cited in the table S1 according to the numbering in the main text; references in positions 127- 138 are mentioned only for table S1.

References

9. Bartlett, C.R., O'Brien, L.B. & Wilson, S.W. A review of the planthoppers (Hemiptera: Fulgoroidea) of the United States. *Mem Am Entomol Soc.* **50,** 1–287 (2014

27. Asche, M. Zur Phylogenie der Delphacidae Leach, 1815 (Homoptera: Cicadina: Fulgoromorpha). *Marburger Ent. Publ*. **1,** 1–398 (1985b); (volume 1), **2,** 399–910 (volume 2) (1985b).

28. Asche, M. Vizcayinae, a new subfamily of Delphacidae with revision of *Vizcaya* Muir (Homoptera: Fulgoroidea) – a significant phylogenetic link. *Bishop Mus. Occas. Papers*, **30,** 154–187 (1990).

29. Emeljanov, A.F. K voprosu o sistemie i filogenii sem. Delphacidae (Homoptera, Cicadina) c uchetom lichinochnykh priznakov. *Entomol. Obozr.* **74** (4), 780–794 (1995). Translated in to English as: Yemel'yanov, A.F. 1996. On the question of the classification and phylogeny of the Delphacidae (Homoptera, Cicadina), with reference to larval characters. *Entomol. Rev.* **75,** 134–150 (1996).

62. Metcalfe, J.R. Studies on the biology of the sugarcane pest *Saccharosydne saccharivora* Homoptera Delphacidae. *Bull. Entomol. Res.* **59** (3), 393–408 (1969[1968]). https://doi.org/10.1017/S0007485300003370

63. Wilson, S.W. & McPherson, J.E. Ontogeny of the tibial spur in *Megamelus davisi* (Homoptera: Delphacidae) and its bearing on delphacid classification. *Great Lakes Entomol.* **14** (1), 49–50 (1981b). https://scholar.valpo.edu/tgle/vol14/iss1

64. Liang, A.-P. New taxa of Vizcayinae (Hemiptera: Auchenorrhyncha: Delphacidae), including a remarkable new genus from China. *J. Nat. Hist.* **36**, 601–616 (2002). https://doi.org/10.1080/00222930110062327

65. Bartlett, C.R. & Webb, M.D. The planthopper genus *Spartidelphax*, a new segregate of Nearctic *Delphacodes* (Hemiptera, Delphacidae). *ZooKeys* **453,** 19–36 (2014). https://doi.org/10.3897/zookeys.453.8369

71. Mora, R., Retana, A. & Espinoza A. M. External morphology of *Tagosodes orizicolus* (Homoptera: Delphacidae) revealed by Scanning Electron Microscopy. *Ann. Entomol. Soc. Am.* **94** (3), 438–448 (2001). https://doi.org/10.1603/0013-8746(2001)094[0438:EMOTOH]2.0.CO;2

76. Donaldson, J.F. Revision of the genus *Notuchus* Fennah (Homoptera: Fulgoroidea: Delphacidae). *J. Austral. ent. Soc.* **18**, 181–185 (1979).

78. Hoch, H., Asche, M., Burwell, C., Monteith, G.M. & Wessel, A. Morphological alteration in response to endogeic habitat and ant association in two new planthopper species from New Caledonia (Hemiptera: Auchenorrhyncha: Fulgoromorpha: Delphacidae). *J. Nat. Hist*. **40** (32), 1867–886 (2006). http://dx.doi.org/10.1080/00222930601046576

83. Asche, M. A new subfamily, genus and species of Delphacidae from South America: Plesiodelphacinae subfam. nov., *Plesiodelphax guayanus* gen. et spec. nov. (Homoptera Fulgoroidea). *Marburger Ent. Publ.* **1** (10), 219–240 (1985a).

84. Asche, M., Hayashi, M. & Fujinuma, S. Enigmatic distribution: first record of a hitherto New World planthopper taxon from Japan (Hemiptera, Fulgoroidea, Delphacidae, Plesiodelphacinae). *Dtsch. Entomol. Z.* **63** (1), 75–88 (2016). https://doi.org/10.3897/dez.63.7178

87. Asche, M. & Emeljanov, A.F. Review of the Neotropical genus *Sparnia* Stål (Hemiptera, Fulgoroidea: Delphacidae). *Entomol. Rev.* **96** (9), 1209–1233 (2016). https://doi.org/ 10.1134/S0013873816090062

88. Susilo, F.X. Swibawa, I G., Indriyati, Hariri, A.M., Purnomo, Hasibuan, R., Wibowo, L., Suharjo, R., Fitriana, Y., Dirmawati, S.R., Solikhin, Sumardiyono, Rwandini, R.A., Sembodo, D.R., & Suputa. The white-bellied planthopper (Hemiptera: Delphacidae) infesting corn plants in South Lampung, Indonesia. *J. Hama penyakit tumbuh. trop.* **17** (1), 96–103 (2017). https://doi.org/10.23960/j.hptt.11796-103

101. Caldwell, J.S. & Martorell, L.F. Review of the auchenorynchous Homoptera of Puerto Rico. Part II. The Fulgoroidea except Kinnaridae. *J. Agr. U. Puerto Rico.* **34** (2), 133–269 (1951).

114. Remes Lenicov, A.M.M. de & Walsh, G.C. A new genus and species of Delphacini (Hemiptera: Fulgoromorpha: Delphacidae) associated with hydrophytic plants in Argentina. *Fla. Entomol.* **96** (4), 1350–1358 (2013).

116. Sosa, A., Remes Lenicov, A.M.M. de & Mariani, R. Species of *Megamelus* (Hemiptera: Delphacidae) Associated with Pontederiaceae in South America. *Ann. Entomol. Soc. Am.* **100** (6), 798–809 (2007). https://doi.org/10.1603/0013-8746(2007)100 [798:SOMHDA]2.0.CO;2

127. https://sites.udel.edu/ planthoppers/north- america/north- american-delphacidae

128. Fennah, R.G. A cavernicolous new species of Notuchus from New Caledonia (Homoptera: Fulgoroidea: Delphacidae). *Rev. Suisse Zool.* **87**(3), 757-759 (1980).

129. Crawford, D. A contribution toward a monograph of the homopterous insects of the family Delphacidae of North and South America. *Proc. U. S. Natl. Mus.* **46** (2041), 557- 640 (1914). https://doi.org/10.5479/si.00963801.46-2041.557

130. Fennah, R.G. Delphacidae from Madagascar and the Mascarene Islands (Homoptera: Fulgoroidea). *Trans. ent. Soc. Lond.* **116**, 131–150 (1964).

131. Hou, X-H. &Chen, X-S. Revision of the planthopper genus *Nycheuma* Fennah (Hemiptera, Fulgoromorpha, Delphacidae). *ZooKeys* **462**, 47–57 (2014). doi: 10.3897/zookeys.462.6657

132 Bellis, G.A. & Donaldson, J.F. Reassessment of some of Kirkaldy's Australian species of Delphacini (Hemiptera: Delphacidae: Delphacinae). *Austral entomol* **55**, 247–260(2016).

133. Otero, M. & Bartlett, C.R. A New Species of *Abbrosoga* (Hemiptera: Fulgoroidea: Delphacidae), An Endemic Puerto Rican Planthopper Genus, with an Updated Checklist of the Delphacidae of Puerto Rico. *Zootaxa* **4563** (2), 372-386(2019). https://doi.org/10.11646/zootaxa.4563.2.10

134. Wilson, S.W. & Tsai, J.H. Descriptions of adults and nymphs of the taro planthopper, *Tarophagus proserpina taiwanensis* ssp. n. from Taiwan (Homoptera: Delphacidae). *Pan-Pac. Entomol.* **64** (1), 53–61(1988).

135. E. A. Heinrichs, Chemical control of the Brown Planthopper. Entomologist, Entomology Department, International Rice Research Institute, Los Baños, Philippines. https://www.researchgate.net/publication/328366128_The_brown_planthopper_threat_to_rice_production_in_Asia/link/5bc8bbc092851cae21af073d

136. Mariani, R. & Remes Lenicov, A.M.M. de. A new species of *Sogatella* (Hemiptera: Delphacidae) from temperate Argentina. *Rev. Bras. Entomol.* **62**, 77–81 (2018). https://doi.org/10.1016/j.rbe.2017.12.001

137. Bartlett, C.R. & Kunz, G.A. New genus and species of delphacid planthopper (Hemiptera: Fulgoroidea: Delphacidae) from Central America with a preliminary regional species list.Zootaxa

3946 (4), 510-518 (2015). doi: 10.11646/zootaxa.3946.4.2.

138. Campodonico, J.F. *Astatometopon sakakibarai* gen. & sp. nov., a montane planthopper from Chile (Hemiptera: Fulgoroidea: Delphacidae). *Acta Ent. Mus. Nat. Pra.* **57** (1), 1–10 (2017).https://doi.org/10.1515/aemnp-2017-0053
